# Histone demethylase KDM4D cooperates with NFIB and MLL1 complex to regulate adipogenic differentiation of C3H10T1/2 mesenchymal stem cells

**DOI:** 10.1038/s41598-020-60049-8

**Published:** 2020-02-20

**Authors:** Jang Hyun Choi, Hansol Lee

**Affiliations:** Department of Biological Sciences, College of Natural Science, Inha University, 100 Inha-ro, Michuhol-gu Incheon, 22212 Korea

**Keywords:** Post-translational modifications, Methylation

## Abstract

The coordinated and sequential actions of lineage-specific transcription factors and epigenetic regulators are essential for the initiation and maintenance of cellular differentiation. We here report KDM4D histone demethylase as a key regulator of adipogenesis in C3H10T1/2 mesenchymal stem cells. The depletion of KDM4D results in impaired differentiation, which can be rescued by exogenous KDM4D, PPARγ, and C/EBPα, but not by C/EBPβ. In addition, KDM4D interacts physically and functionally with both NFIB and MLL1 complex to regulate C/EBPα and PPARγ expression upon adipogenic hormonal induction. Although KDM4D is dispensable for the binding of both NFIB and MLL1 complex to the target promoters, the demethylation of tri-methylated H3K9 by KDM4D is required for NFIB and MLL1 complex to deposit tri-methylated H3K4 and activate PPARγ and C/EBPα expression. Taken together, our data provide a molecular framework for lineage-specific transcription factor and histone modifiers to cooperate in adipogenic differentiation, in which KDM4D removes repressive histone marks at genes with a bivalent chromatin domain and allows NFIB and MLL1 complex to promote the expression of key adipogenic regulators.

## Introduction

The differentiation of mesenchymal stem cells (MSCs) to adipocytes is initiated and maintained by the coordinated actions of signal transduction pathways and a series of transcription programs operating at different stages of differentiation^1–3^. Peroxisome proliferator activator receptor γ (PPARγ) and CCAAT/enhancer-binding protein α (C/EBPα) are in the center of the regulatory network for adipocyte differentiation and play critical roles in the expression of genes associated with adipogenesis and lipid metabolism^4,5^. A number of pro-/anti-adipogenic transcription factors have been identified, which include CCAAT/enhancer binding proteins (C/EBPs), early B-cell factors (EBFs), Krüppel-like factors (KLFs), sterol regulatory element-binding protein-1 (SREBP-1), and nuclear factor I family of transcription factors (NFI)^6–8^. These factors have been shown to regulate the expression or function of PPARγ either directly or indirectly.

Lysine methylation of histones is a prominent posttranslational modification that has been implicated in both the activation and repression of genes depending on the sites and status of modification^9^. Di- and tri-methylation of histone H3 lysine 9 (H3K9) as well as the tri-methylation of histone H3 lysine 27 (H3K27) are recognized as the hallmarks of gene repression^10^, whereas the methylation of histone H3 lysine 4 (H3K4) and lysine 36 (H3K36) are associated with gene activation^11–13^. Histone lysine methyltransferases (KMTs) have been implicated in the regulation of adipogenic differentiation^14^. KMTs mediating the di- and tri-methylation of H3K9 act as negative regulators of adipogenesis. G9a and SUV39H1 inhibit adipogenesis by repressing the expression of key adipogenic regulators, PPARγ and C/EBPα, respectively^15,16^. In addition, SETDB1 has recently been reported to contribute to the establishment of the H3K4me3/H3K9me3 bivalent chromatin domain, which is believed to make MSCs and preadipocytes paused for adipocyte differentiation^17^. In contrast, KMTs responsible for the methylation of H3K4 is associated primarily with the positive regulation of adipogenesis. MLL3 (KMT2C) and MLL4 (KMT2D), which are members of the MLL family responsible for the mono- and di-methylation of H3K4, are required for the expression of PPARγ and C/EBPα in cells as well as adipogenesis *in vivo*^18,19^. On the other hand, the function of MLL1/MLL2 (KMT2A/KMT2B) in adipogenesis, which mediates tri-methylation of H3K4^20^, has not been studied in detail and remains elusive.

In addition to KMTs, members of histone demethylase (KDM) family of proteins have been suggested to be regulators of adipogenesis. Lysine-specific demethylase 1 (LSD1) promotes adipogenesis by demethylating H3K4 on the promoter regions of the Wnt signaling components^21^, whereas H3K27 specific histone demethylase JMJD3 (KDM6B) is essential for the expression of brown fat (BAT)-selective genes^22^. The KDM4/JMJD2 family proteins are JmjC domain-containing histone demethylases responsible for the removal of H3K9me3 as well as H3K36me3^23,24^ and have been implicated in the regulation of adipogenesis^25–28^. KDM4A plays critical role in the PPARγ-mediated regulation of a specific transcription program, and KDM4B has been shown to interact with C/EBPβ to promote mitotic clonal expansion during the differentiation of 3T3-L1 preadipocytes^26,27^. On the other hand, the roles of KDM4D in adipogenic differentiation, which targets H3K9 only not H3K36, have not been defined yet^29^.

KMTs and KDMs are often found in the same complex, and it is believed that they cooperate to regulate gene expression^30,31^. Both MLL3/MLL4 and an H3K27 specific histone demethylase, UTX, are associated with the Pax transactivation domain-interacting protein (PTIP), and they cooperate to regulate the expression of PPARγ and C/EBPα in adipogenic differentiation^32^. In addition, MLL2 and KDM4B cooperate in estrogen receptor α (ERα)-regulated transcription^31^. In this report, we have identified KDM4D as a critical regulator of the adipogenic differentiation of C3H10T1/2 MSCs. In addition, KDM4D interacts physically with MLL1 complex as well as the NFIB transcription factor, and that all of these three factors are required for adipogenesis. RNA-seq analysis and chromatin immunoprecipitation analysis support the idea that KDM4D, MLL1, and NFIB function as a complex and promote the expression of adipogenic genes including PPARγ and C/EBPα. Finally, we demonstrate coordinated actions of KDM4D, NFIB, and MLL1 complex in the expression of PPARγ and C/EBPα, in which KDM4D functions as a ‘gate-opener’ by removing the repressive H3K9me3 and allows NFIB and MLL1 complex to mediate the tri-methylation of H3K4 and activate the transcription of PPARγ and C/EBPα.

## Results and Discussion

### KDM4D is required for the adipogenic differentiation of C3H10T1/2 mesenchymal stem cells

**Figure 1:**
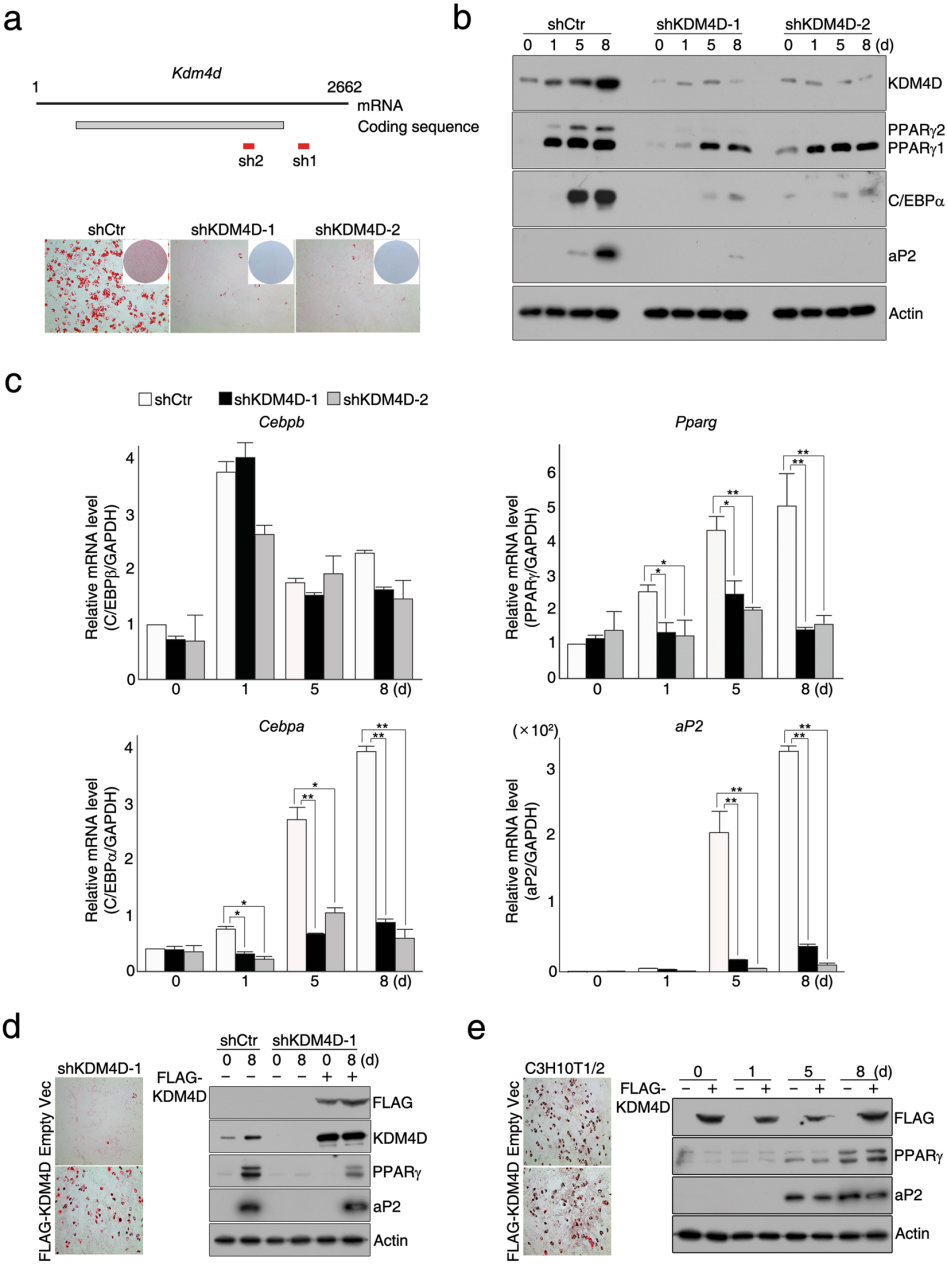
KDM4D is required for the adipogenic differentiation of C3H10T1/2 mesenchymal stem cells. (**a–c**). Knockdown of KDM4D by the shRNA inhibits the adipogenesis of C3H10T1/2 cells. C3H10T1/2 cells and two independently established KDM4D-knockdowned cells (shKDM4D-1 and shKDM4D-2) were grown to confluence and adipogenic differentiation was induced by MDI hormonal treatment. (**a**) Schematic diagram depicting the sites of *Kdm4d* mRNA targeted by shRNA (upper). Sh1 targets 3′-UTR (shKDM4D-1) and Sh2 targets the coding region of *Kdm4d* mRNA (shKDM4D-2). Oil Red O staining at day eight after the induction of differentiation. The insets present plate images after ORO staining (lower). (**b**) Immunoblot analysis of KDM4D and key adipogenic markers before and after induction of differentiation. The total extracts were prepared from the cells at indicated time points (day 0; before MDI treatment) and subjected to immunoblot analysis to detect KDM4D, PPARγ, C/EBPα, and aP2. Actin was used as a loading control. (**c**) RT-qPCR analysis of adipogenic gene expression before and after the induction of differentiation. The total RNAs were isolated from control, shKDM4D-1, and shKDM4D-2 cells at the indicated time points, and the relative mRNA levels of *Cebpb*, *Pparg*, *Cebpa*, and *aP2* were measured by RT-qPCR. The mRNA levels were first normalized to the mRNA level of *GAPDH* and the data are presented as the ratio of the mRNA level at each time point (after induction) to the mRNA level at day 0 (before induction). Quantitative PCR (qPCR) data are representative of at least three independent experiments and are presented as mean ± SD. *p < 0.05; **p < 0.01. **(d)** Rescue of differentiation by exogenous FLAG-KDM4D. The control cells and shKDM4D-1 cells, in which shRNA targets 3′ UTR of *Kdm4d*, were infected with a retrovirus expressing empty vector or FLAG-KDM4D. (Left) Oil Red O staining at day eight after the induction of differentiation. (Right) Immunoblot analysis of KDM4D (with α-FLAG and α-KDM4D), PPARγ, and aP2 before (day 0) and after (day 8) induction of differentiation. Actin was used as a loading control. **(e)** Overexpression of KDM4D had no significant effect on the adipogenic differentiation of C3H10T1/2 MSCs. The cells were infected with the retrovirus expressing empty vector or FLAG-KDM4D, followed by the induction of adipogenic differentiation. (Left) Oil Red O staining at day eight after the induction of differentiation. (Right) The total extracts were prepared from the cells at the indicated time points and subjected to immunoblot analysis of KDM4D, PPARγ, and aP2. Actin was used as a loading control. The images of immunoblot analysis (b,d,e) were cropped from different gels due to similar molecular weight but all panels in each figure are from the same experiment. Unprocessed images are provided in the supplementary information.

During the adipogenic differentiation of C3H10T1/2 mesenchymal stem cells (MSCs), the levels of *Kdm4d* mRNA and KDM4D protein increased gradually, which is similar to other members of the *Kdm4* family of genes and is correlated with the expression patterns of PPARγ and C/EBPα (Supplementary Fig. S1a,b). To investigate whether KDM4D is required for adipogenesis, two stable cell lines (shKDM4D-1 and shKDM4D-2), in which endogenous KDM4D is decreased by shRNAs (Supplementary Fig. S1c), and their ability to differentiate into adipocytes upon MDI induction was tested. As shown in Fig. 1a, depletion of endogenous KDM4D severely hampered the adipogenic potential of C3H10T1/2 cells, which is evident by the little or no accumulation of lipid droplets even at day 8 after MDI induction. In addition, whereas significant increases in the level of key adipogenic markers such as PPARγ (both PPARγ1 and PPARγ2), C/EBPα, and aP2 were observed in control cells (shCtr) during differentiation, activation of PPARγ2, C/EBPα, and aP2 were significantly inhibited and activation of PPARγ1 was markedly decreased in both shKDM4D-1 and shKDM4D-2 cells after induction of adipogenic differentiation (Fig. 1b,c). In particular, the expression of C/EBPβ, a well-known regulator of PPARγ and C/EBPα expression, was not affected by KDM4D knockdown (Fig. 1c). We next examined whether KDM4D lacking 3′-UTR could rescue adipogenic differentiation in shKDM4D-1 cells because the shRNA used for KDM4D depletion in shKDM4D-1 cells targets 3′-UTR of *Kdm4d* mRNA. As expected, replenishing the cellular KDM4D pool with exogenous FLAG-KDM4D lacking 3′-UTR successfully recovered the adipogenic potential in shKDM4D-1 cells (Fig. 1d and Supplementary Fig. S1d). In contrast to the inhibitory effects of KDM4D depletion on adipogenic differentiation, the overexpression of FLAG-KDM4D in C3H10T1/2 MSCs had no significant effects on promoting adipogenic differentiation (Fig. 1e and Supplementary Fig. S1e). These findings suggest that KDM4D is necessary but not sufficient for adipogenic differentiation in C3H10T1/2 MSCs.

### Exogenous expression of PPARγ or C/EBPα rescues adipogenic differentiation in KDM4D-depleted cells

**Figure 2:**
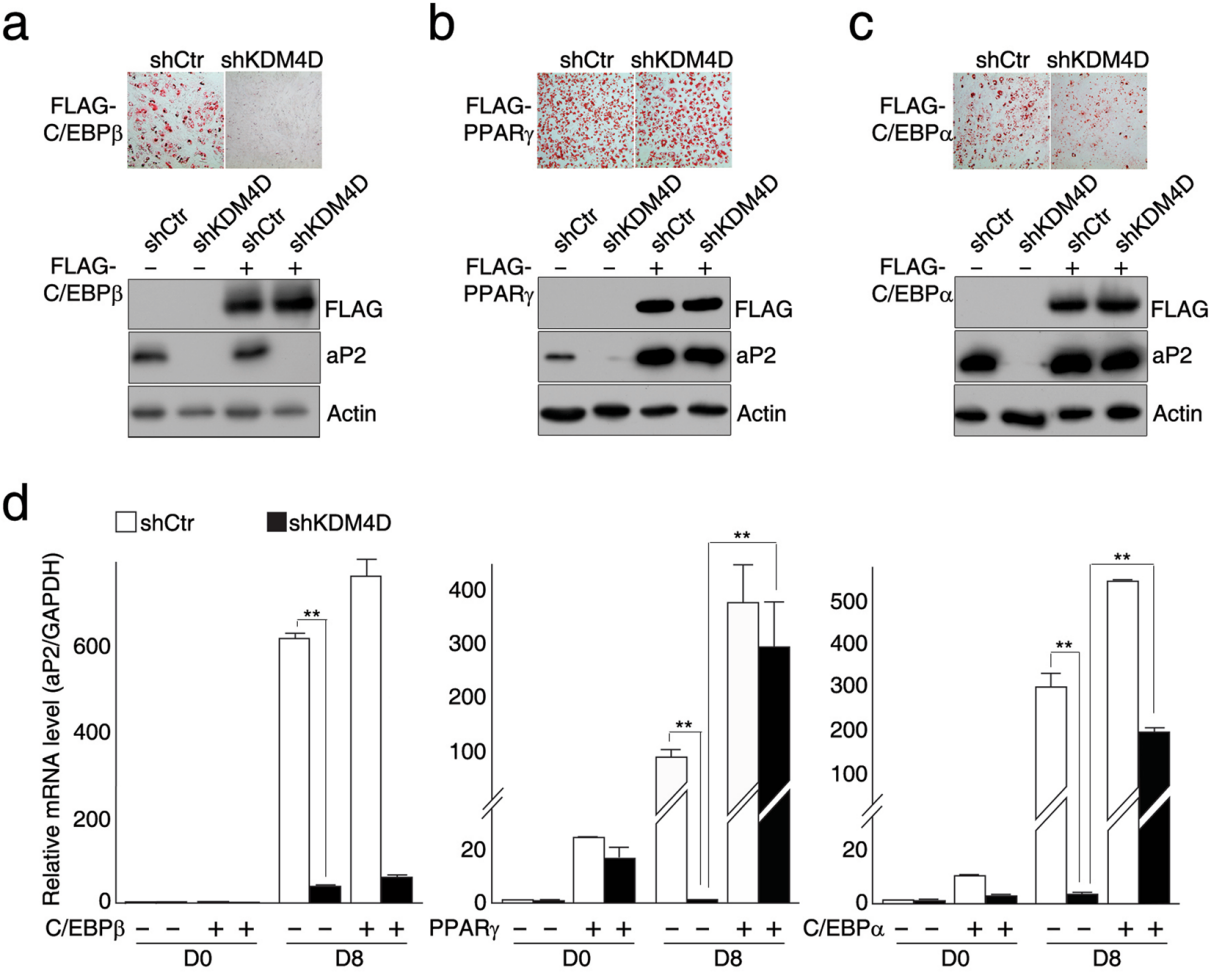
Exogenous expression of PPARγ or C/EBPα rescues adipogenic differentiation in KDM4D-depleted cells. The control and shKDM4D cells were infected with retrovirus expressing empty vector or FLAG-tagged C/EBPβ (**a**), PPARγ (**b**), or C/EBPα (**c**), followed by the induction of differentiation for eight days. **(a)** Exogenous expression of FLAG-C/EBPβ does not rescue adipogenic differentiation in shKDM4D cells. Oil Red O staining at day eight after the induction of differentiation (upper) and immunoblot analysis of FLAG-C/EBPβ and aP2 (lower). **(b,c)** Exogenous expression of FLAG-PPARγ (**b**) or FLAG-C/EBPα (**c**) rescues adipogenic differentiation in shKDM4D cells. Oil Red O staining at day 8 after induction of differentiation (upper) and immunoblot analysis of FLAG-PPARγ (**b**), FLAG-C/EBPα (**c**), and aP2 (lower). **(d)** RT-qPCR analysis of *aP2* in the control and shKDM4D cells infected with the retrovirus expressing empty vector or FLAG-C/EBPβ (left), FLAG-PPARγ (middle) or FLAG-C/EBPα (right). The mRNA levels of *aP2* were first normalized to the mRNA level of *GAPDH*, and the data are presented as the ratio of mRNA level at each time point to the mRNA level in the control cells at day 0 (before induction). The quantitative PCR data are representative of at least three independent experiments and presented as mean ± SD. **p < 0.01. The images of immunoblot analysis (**a–c**) were cropped from different gels because of similar molecular weight. All panels in each figure are from the same experiment. Unprocessed images are provided in the supplementary information.

Wnt signaling plays critical roles in the fate determination of mesenchymal stem cells (MSCs). The activation of Wnt signaling facilitates the osteogenic differentiation of MSCs and its inhibition allows MSCs to differentiate into adipocytes, presumably via activation of C/EBPβ^33^. Because there have been reports suggesting that KMT and KDM are involved in adipogenic differentiation through regulating the expression of Wnt ligands^21,34^, we examined whether KDM4D regulates the expression of Wnt ligands (Supplementary Fig. S2). Interestingly, neither the overexpression of KDM4D nor the depletion of endogenous KDM4D led to significant changes in the expression of Wnt ligands, such as *Wnt1*, *Wnt6*, *Wnt10a*, and *Wnt10b*. The factors that promote adipogenesis were assessed further to determine if they could override the effects of KDM4D depletion in shKDM4D cells. The overexpression of FLAG-C/EBPβ was not sufficient for restoring adipogenic differentiation in shKDM4D cells (Fig. 2a,d). In particular, the exogenous expression of PPARγ alone was enough to override the inhibitory effects of KDM4D depletion and exogenous expression of C/EBPα, and to a lesser extent, resulted in meaningful recovery of the adipogenic potential in shKDM4D cells (Fig. 2b–d). These results suggest that the inhibition of adipogenic differentiation by KDM4D depletion resulted from defects in the transcription activation of *Pparg* and *Cebpa*.

### KDM4D physically interacts with NFIB and MLL1 complex

**Figure 3:**
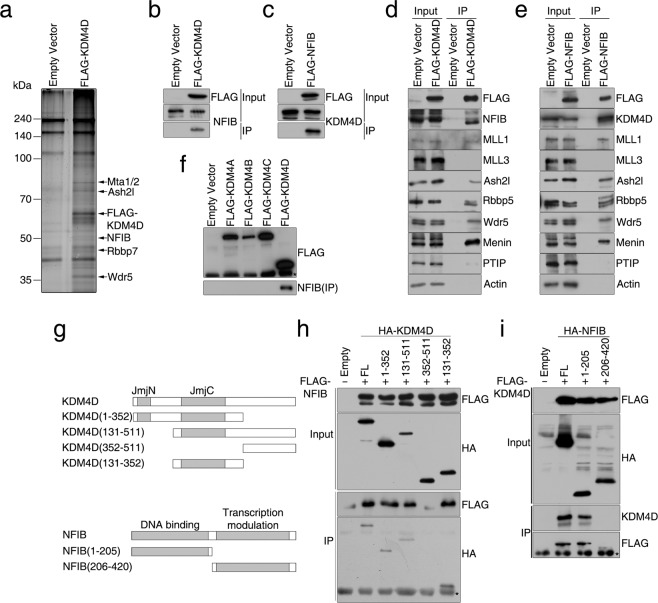
KDM4D interacts with NFIB transcription factor and MLL1 H3K4 methyltransferase complex. (**a**) KDM4D was co-purified with NFIB, components of MLL complex, and components of NuRD remodeling complex. C3H10T1/2 cells were infected with the retrovirus expressing empty vector or FLAG-KDM4D and nuclear extracts were prepared as described in methods. KDM4D interacting proteins were purified with FLAG-M2 agarose and subjected to mass spectrometric analysis. Shown is a silver staining image of FLAG-KDM4D and co-purified proteins. (**b,c**) KDM4D interacts with NFIB in C3H10T1/2 cells. The cells were infected with the retrovirus expressing empty vector, FLAG-KDM4D (**b**) or FLAG-NFIB (**c**). The protein extracts were immunoprecipitated with FLAG-M2 agarose and the interactions were confirmed by immunoblot analysis using α-NFIB in (**b**) and α-KDM4D in (**c**). **(d,e)** KDM4D and NFIB interact with MLL1 histone methyltransferase complex in C3H10T1/2 cells. The cells were infected with the retrovirus expressing empty vector, FLAG-KDM4D (**d**), or FLAG-NFIB (**e**). The protein extracts were immunoprecipitated with FLAG-M2 agarose, and the interactions were examined by immunoblot analysis using the indicated antibodies. Actin was used as a loading control. **(f)** KDM4D, but not other members of the KDM4 family of proteins, interacts with NFIB. C3H10T1/2 cells were infected with the retrovirus expressing empty vector or indicated FLAG-KDM4 proteins (KDM4A - KDM4D). The protein extracts were immunoprecipitated with FLAG-M2 agarose, followed by immunoblot analysis to determine the interaction with endogenous NFIB. The asterisk indicates non-specific band. (**g**) Schematic diagrams of KDM4D (upper), NFIB (lower), and their deletion mutants. JmjN and JmjC domains of KDM4D (upper) and N-terminal DNA binding domain and transcription modulation domain of NFIB (lower) are shown as gray boxes. (**h**) JmjC domain of KDM4D mediates the interaction with NFIB. Human 293 T cells were co-transfected with plasmids containing full-length FLAG-NFIB and indicated HA-KDM4D (FL; full-length, numbered; deletion mutants). Half of the protein extracts were immunoprecipitated with FLAG-M2 agarose and the other half were immunoprecipitated with α-HA antibody. The interactions between full-length NFIB and KDM4D (and its deletion mutants) were determined by immunoblot analysis using α-HA (for FLAG IP) and α-FLAG (for HA IP). The asterisk indicates the immunoglobulin light chain band. **(i)** N-terminal domain of NFIB is required for the interaction with KDM4D. Human 293 T cells were co-transfected with plasmids containing full-length FLAG-KDM4D and indicated HA-NFIB constructs (FL; full-length, numbered; deletion mutants). The protein extracts were immunoprecipitated with α-HA antibody. Interactions between the full-length KDM4D and NFIB (and its deletion mutants) were determined by immunoblot analysis using α-FLAG and α-KDM4D. The asterisk indicates the immunoglobulin heavy chain band. The images of immunoblot analysis shown were cropped from different gels to optimize separation (MLL proteins) and distinguish the proteins with similar molecular weight. Unprocessed images are provided in the supplementary information.

Because the overexpression of KDM4D alone is insufficient for promoting adipogenesis in C3H10T1/2 cells (Fig. 1e), the function of KDM4D might be mediated by an interaction with other cellular factors. A recent report showing that KDM4B, a member of the KDM4 family, interacts with C/EBPβ to regulate adipogenic differentiation also supports the idea of protein-protein interaction being the key factor in the function of KDM4D^26^. Therefore, we examined whether KDM4D interacts with the adipogenic transcription factors, such as C/EBPβ, PPARγ, and C/EBPα by a co-immunoprecipitation assay, but no evidence to support the physical interaction of KDM4D with those adipogenic regulators was found (data not shown). Next, mass spectrometry of proteins co-purified with FLAG-KDM4D in C3H10T1/2 cells was carried out and a list of KDM4D interacting protein candidates was identified, which included NFIB, Wdr5, Ash2l, and members of the NuRD nucleosome remodeling complex; namely Mta1/2 and Rbbp7 (Fig. 3a). We were particularly interested in NFIB transcription factor and MLL complex, Wdr5 and Ash2l containing H3K4 methyltransferases, because both of them have been implicated in the regulation of adipogenic differentiation^7,18,19^. The interaction of KDM4D with NFIB in C3H10T1/2 cells as well as in human 293 T cells was first verified (Fig. 3b,c and Supplementary Fig. S3a). In addition, co-immunoprecipitation assays using FLAG-M2 agarose showed that both KDM4D and NFIB interact with the core components of MLL complex; Ash2l, Rbbp5, and Wdr5 (Fig. 3d,e and Supplementary Fig. S3b,c). Surprisingly, both KDM4D and NFIB interacted with MLL1 and Menin, a member of the MLL1/2 complex, but not with MLL3 and MLL3/4 - specific PTIP in C3H10T1/2 cells (Fig. 3d,e). In addition, other members of the KDM4 family of histone demethylases, KDM4A, KDM4B, and KDM4C, did not have any meaningful interactions with NFIB and MLL1 (Fig. 3f), suggesting that KDM4D interacts specifically with NFIB and MLL1/2 complex. The protein domain of KDM4D required for the interaction with NFIB is contained in the region including the JmjC domain, corresponding to residues 131 to 352, because a deletion of it disrupted the interaction and this region alone was sufficient for the interaction with NFIB (Fig. 3g,h). Conversely, an analysis of truncated NFIBs showed that the N-terminal region (amino acids 1–205) containing the MH1 domain is necessary and sufficient for the interaction with KDM4D (Fig. 3g,i). The same regions of KDM4D and NFIB were also required for the interaction with Wdr5, a core component of MLL complexes (Supplementary Fig. S3d,e). Overall, these results suggest that KDM4D, NFIB, and MLL1/2 complex might form a tertiary complex in C3H10T1/2 MSCs and possibly cooperate in the regulation of adipogenic differentiation.

### KDM4D, NFIB, and MLL1 complex commonly target *Pparg* and *Cebpa* to regulate adipogenic differentiation

**Figure 4:**
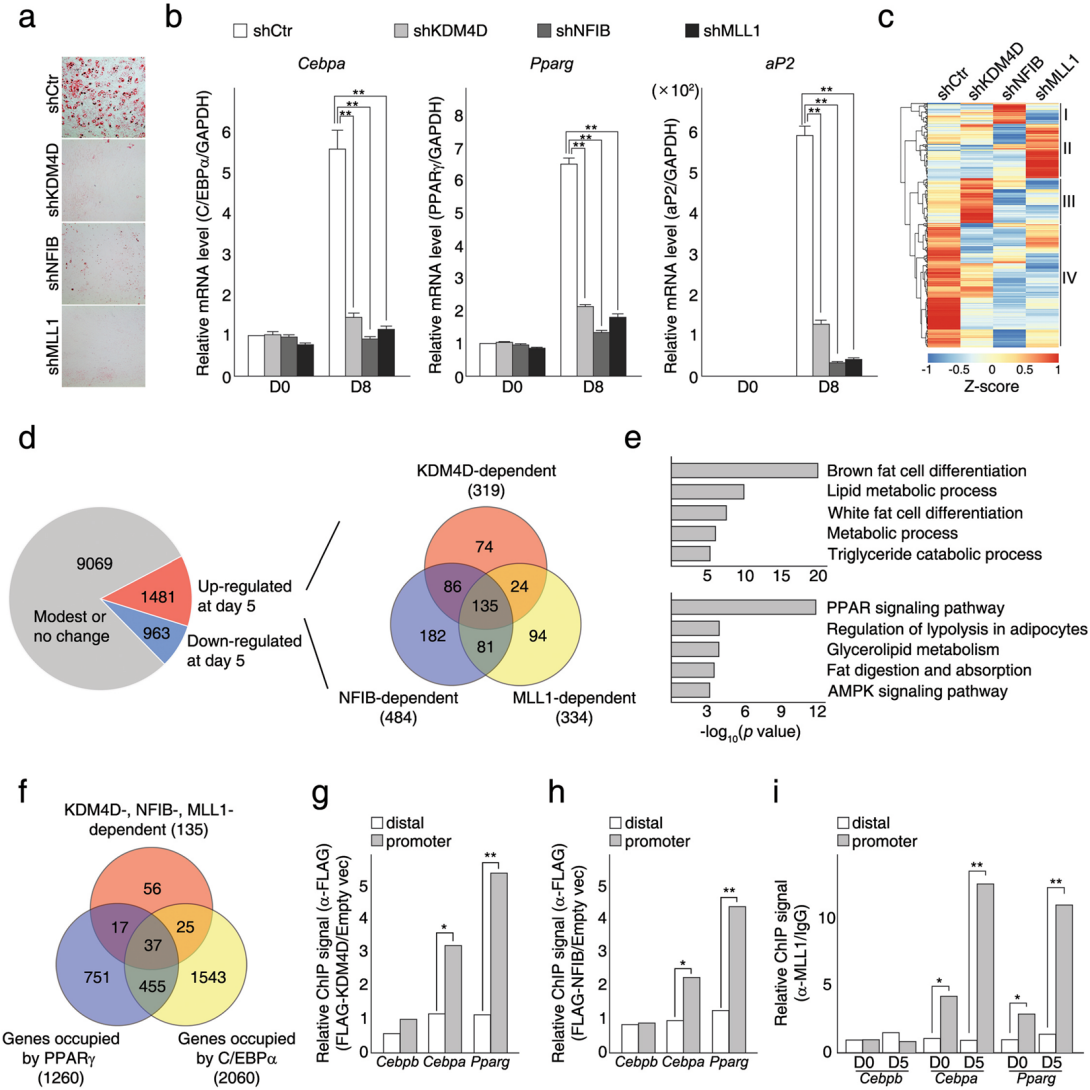
KDM4D, NFIB, and MLL1 complex work together in regulating adipogenic differentiation. (**a,b**) NFIB and MLL1 are required for the adipogenic differentiation of C3H10T1/2 cells. The control and three different KD cells (shKDM4D, shNFIB and shMLL1) were grown to confluence, and adipogenic differentiation was induced by MDI hormonal treatment. (**a**) Oil Red O staining at day eight after the induction of differentiation. (**b**) RT-qPCR analysis of adipogenic gene expression before and after the induction of differentiation. The total RNAs were isolated from the control, shKDM4D, shNFIB, and shMLL1 cells before (day 0) and after (day 8) the induction of differentiation. The relative mRNA levels of *Pparg*, *Cebpa*, and *aP2* were measured by RT-qPCR. The mRNA levels were normalized to the mRNA level of *GAPDH* and the data are presented as the ratio of the mRNA level at each time point to the mRNA level in the control cells at day 0. Quantitative PCR data are representative of at least three independent experiments and presented as mean ± SD. **p < 0.01. (**c–f**) KDM4D, NFIB, and MLL1 are required for adipogenic gene expression. The cells were collected before (day 0) and after (day 5) the induction of differentiation for RNA-seq analysis. (**c**) RNA-seq heatmap depicting the changes in expression of the genes (upregulated during adipogenic differentiation of C3H10T1/2 cells) in the control, shKDM4D, shNFIB, and shMLL1 cells. The color intensity scale was included at the bottom of the heatmap. The threshold for up-regulation is 2.0 fold. (**d**) Schematic diagram of the identification of KDM4D-, NFIB-, and MLL1-dependent genes (left) and Venn diagram of the genes affected by KDM4D, NFIB, and MLL1 depletion (lower than the control cells at day 5). (**e**). Gene ontology (GO) analysis (upper) and KEGG pathway analysis (lower) of the genes defined as commonly affected genes in (**d**). (**f**) Venn diagram of the genes identified as KDM4D/NFIB/MLL1-dependent and direct targets of PPARγ and C/EBPα. (**g,h**) KDM4D and NFIB bind to the promoter of *Cebpa*, and *Pparg* genes. C3H10T1/2 cells were infected with a retrovirus expressing empty vector or FLAG-KDM4D (**g**) and FLAG-NFIB (**h**). Chromatins prepared from the cells were precipitated with FLAG-M2 agarose, and quantitative PCR (qPCR) analysis was performed to assess the binding to the promoter and distal region of the indicated genes. For the relative ChIP signal, the % input was calculated for each sample, and data are presented as the ratio of the % input in FLAG-KDM4D-infected cells to the % input in the control cells. (**i**) MLL1 binds to the promoter of *Cebpa*, and *Pparg* genes. Chromatins prepared from the cells before (day 0) and after (day 5) the induction of differentiation were precipitated with IgG or α-MLL1 antibody. qPCR analysis was performed as in (**g,h**). For the relative ChIP signal, the % input was calculated for each sample and data are presented as the ratio of the % input (α-MLL1) to the % input (IgG). Quantitative PCR data shown in (**g**–**i**) are representative of at least three independent experiments and are presented as mean ± SD. *p < 0.05; **p < 0.01.

To confirm that KDM4D, NFIB, and MLL1 complex are involved in the regulation of adipogenesis, particularly in the transcription activation of *Pparg* and *Cepba*, stable cell lines expressing shRNAs against NFIB, MLL1, or Ash2l in C3H10T1/2 MSCs were established and tested for adipogenic differentiation upon MDI induction (Fig. 4a and Supplementary Fig. S4a–c). We chose MLL1 over MLL2 because it has been reported that MLL2 interacts with KDM4B in other contexts^31^, and KDM4B cooperates with C/EBPβ in 3T3-L1 preadipocytes^26^. As expected, the depletion of NFIB in MSCs (shNFIB) resulted in inhibited differentiation. Importantly, the knockdown of MLL1 (shMLL1) led to severely hampered adipogenic differentiation upon MDI induction, which has not been previously reported (Fig. 4a). Consistently, RT-qPCR analysis confirmed that depletion of NFIB or MLL1 leads to defects in the transcription activation of *Pparg* and *Cebpa* but not *Cebpb* upon MDI induction, which is similar to the effects of KDM4D depletion (Fig. 4b). In addition, the knockdown of Ash2l (shAsh2l) also led to impaired adipogenic differentiation (Supplementary Fig. S4c), showing that both NFIB and MLL1 complex are essential for the adipogenic differentiation of C3H10T1/2 MSCs.

RNA-seq analysis of eight different samples (RNAs isolated from C3H10T1/2, shKDM4D, shMLL1, and shNFIB cells at day 0 and day 5) was next conducted to determine if the genes affected by KDM4D depletion overlapped with those deregulated by NFIB or MLL1 knockdown, particularly those associated with adipogenic differentiation, such as *Pparg* and *Cebpa* (log2 FC > 1 and FDR < 0.05). We first identified 1481 up-regulated and 963 down-regulated genes during the adipogenic differentiation of C3H10T1/2 MSCs and designated them as adipogenesis-associated genes (AAGs) (Supplementary Data S1). Cluster analysis and DEG (differentially expressed gene) analysis showed that the expression levels of 319, 484, and 334 genes out of 1481 up-regulated AAGs were markedly affected by the knockdown of KDM4D, NFIB, and MLL1, respectively (Fig. 4c,d). Of particular interest, 42.3% (135/319) of the genes affected by KDM4D depletion, which included *Pparg* and *Cebpa*, were also presented in the list of genes affected by NFIB knockdown and MLL1 depletion (Fig. 4d and Supplementary Data S2). GO analysis and KEGG pathway analysis showed that many of these 135 genes are strongly associated with brown fat/white fat cell differentiation (GO:0050873 and GO:0050872), lipid metabolic process (GO:0006629) (Fig. 4e, upper), and importantly, with the PPAR signaling pathway (mmu03320) (Fig. 4e, lower). On the other hand, relatively fewer genes out of 963 down-regulated AAGs were affected by knockdown of KDM4D (64 genes), NFIB (249 genes), and MLL1 (196 genes) (Supplementary Fig. S4d, e). Although 60.9% (39/64) of the genes affected by KDM4D depletion appeared to be common targets of all three proteins, GO and KEGG analysis of those 39 genes showed no obvious link to adipogenic differentiation (Supplementary Data S2). Next, we obtained a list of PPARγ or C/EBPα target genes from the chromatin immunoprecipitation (ChIP)-atlas databases and NCBI GEO database, and compared them with 135 genes identified as common targets of the KDM4D, NFIB, and MLL1 complex (Fig. 4f). Surprisingly, 58.5% of the genes (79/135) were included in the list of direct targets of PPARγ, C/EBPα, or both (Fig. 4f and Supplementary Data S3). Chromatin immunoprecipitation assays confirmed that both FLAG-KDM4D and FLAG-NFIB bind specifically to the promoters of *Pparg* and *Cebpa*, but not to the *Cebpb* promoter (Fig. 4g,h). Consistently, significant enrichments of MLL1 and Ash2l were also found in the same regions (Fig. 4i and Supplementary Fig. S4f). In addition, increases in the level of tri-methylated histone H3 lysine 4 (H3K4me3), as well as decreases in tri-methylated histone H3 lysine 9 (H3K9me3), were observed at the promoters of *Pparg* and *Cebpa* during the adipogenic differentiation of C3H10T1/2 MSCs (Supplementary Fig. S4g,h). Interestingly, the levels of both H3K4me3 and H3K9me3 were significantly higher at the promoter than at the distal region of the genes in undifferentiated MSCs (Supplementary Fig. S4g,h), which is in line with the notion that the bivalent H3K4me3/H3K9me3 chromatin domain exists at the promoter of adipogenic regulators^17^. Overall, these results strongly suggest that the functions of KDM4D as an adipogenic regulator are mediated, at least in part, by the direct regulation of PPARγ and C/EBPα expression, and are possibly involved in resolving the bivalent chromatin domains through interactions with NFIB and MLL1 complex.

### KDM4D is dispensable for interactions between NFIB and MLL1 complex, but MLL1 complex is required for the interaction between KDM4D and NFIB as well as for their binding to the target promoters

**Figure 5:**
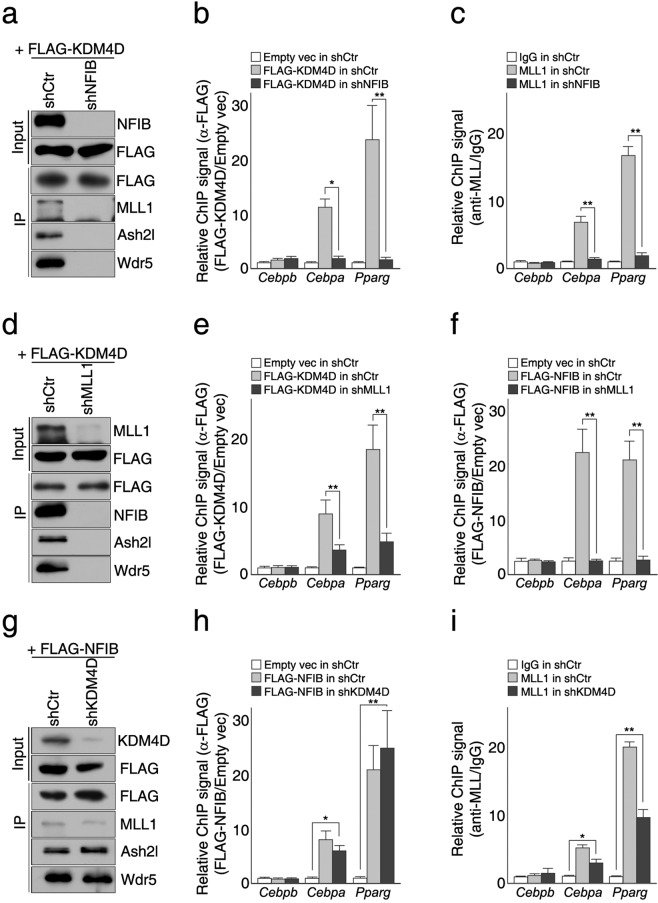
KDM4D requires MLL1 to interact with NFIB but is dispensable for the interaction between NFIB and MLL1 complex as well as their binding to the *Pparg* and *Cebpa* promoters. Control and KD cells (shNFIB, shMLL1, and shKDM4D, respectively) were infected with the retrovirus expressing empty vector or indicated FLAG-tagged proteins. The total protein extracts and chromatins were prepared after the induction of differentiation (day 5), followed by the immunoprecipitation assay and ChIP-qPCR analysis. (**a–c**) NFIB is required for the interaction between KDM4D and MLL1 complex and their binding to the *Pparg* and *Cebpa* promoters. (**a**) Immunoblot analysis of the interaction between FLAG-KDM4D and MLL1 complex in the control and shNFIB cells. (**b,c**) ChIP-qPCR analysis of FLAG-KDM4D (**b**) and MLL1 (**c**) bindings to the *Cebpb*, *Cebpa*, and *Pparg* promoters in the control and shNFIB cells. (**d–f**) MLL1 is necessary for the interaction between KDM4D and NFIB and their binding to the target promoters. (**d**) Immunoblot analysis of the interaction between FLAG-KDM4D and NFIB in the control and shMLL1 cells. (**e,f**) ChIP-qPCR analysis of FLAG-NFIB (**e**) and FLAG-KDM4D (**f**) bindings to the *Cebpb*, *Cebpa*, and *Pparg* promoters in the control and shMLL1 cells. (**g–i**) KDM4D is dispensable for the interaction between NFIB and MLL1 complex and their bindings to the target promoters. (**g**) Immunoblot analysis of the interaction between FLAG-NFIB and MLL1 complex in the control and shMLL1 cells. (**h,i**) ChIP-qPCR analysis of FLAG-NFIB (**h**) and MLL1 (**i**) bindings to the *Cebpb*, *Cebpa*, and *Pparg* promoters in the control and shKDM4D cells. For the relative ChIP signal, the % input was calculated for each sample. Data shown in figures (**b**,**e**,**f**,**h**) were presented as the ratio of the % input in the cells expressing FLAG-KDM4D (**b,f**) or FLAG-NFIB (e and h) to the % input in the control cells infected with retrovirus expressing empty vector. Data shown in figures (**c,i**) were presented as the ratio of the % input (α-MLL1) to % input (IgG) in the control cells. Quantitative PCR data in all figures are representative of three independent experiments and are presented as mean ± SD. *p < 0.05; **p < 0.01. The images of immunoblot analysis shown (**a,d,g**) were cropped from different gels to optimize separation (MLL proteins) and to distinguish proteins with a similar molecular weight. All panels in each figure are from the same experiment. Unprocessed images are provided in the supplementary information.

NFIB is a sequence-specific DNA binding protein that is likely to function as a transcription activator, and both KDM4D and MLL1 complex are histone modifying enzymes associated primarily with transcription activation. Therefore, we next investigated how their functions are related to the protein-protein interaction in the regulation of PPARγ and C/EBPα expression during adipogenic differentiation (Fig. 5). In the cells depleted of NFIB (shNFIB), no physical interactions were observed between exogenous FLAG-KDM4D and MLL1 complex (Fig. 5a). Consistently, no significant enrichment of FLAG-KDM4D or MLL1 was found at the promoters of *Pparg* and *Cebpa*, suggesting that NFIB is essential for both histone modifying enzymes to be recruited to the target genes (Fig. 5b,c). Similarly, FLAG-KDM4D did not interact with NFIB and their binding to the target promoters decreased significantly in shMLL1 cells and in shAsh2l cells, (Fig. 5d–f and Supplementary Fig. S5a–c). Surprisingly, the interaction between NFIB and MLL1 complex was unaffected by the knockdown of KDM4D and both FLAG-NFIB and MLL1 could bind to the *Pparg* and *Cebpa* promoters in cells depleted of KDM4D (Fig. 5g–i). This suggests that while MLL1 complex is required for the interaction between KDM4D and NFIB as well as their binding to the target promoters, KDM4D itself is dispensable for both NFIB and MLL1 complex to bind to the target promoters.

### KDM4D coordinates H3K4me3/H3K9me3 to regulate adipogenic differentiation of C3H10T1/2 mesenchymal stem cells

**Figure 6:**
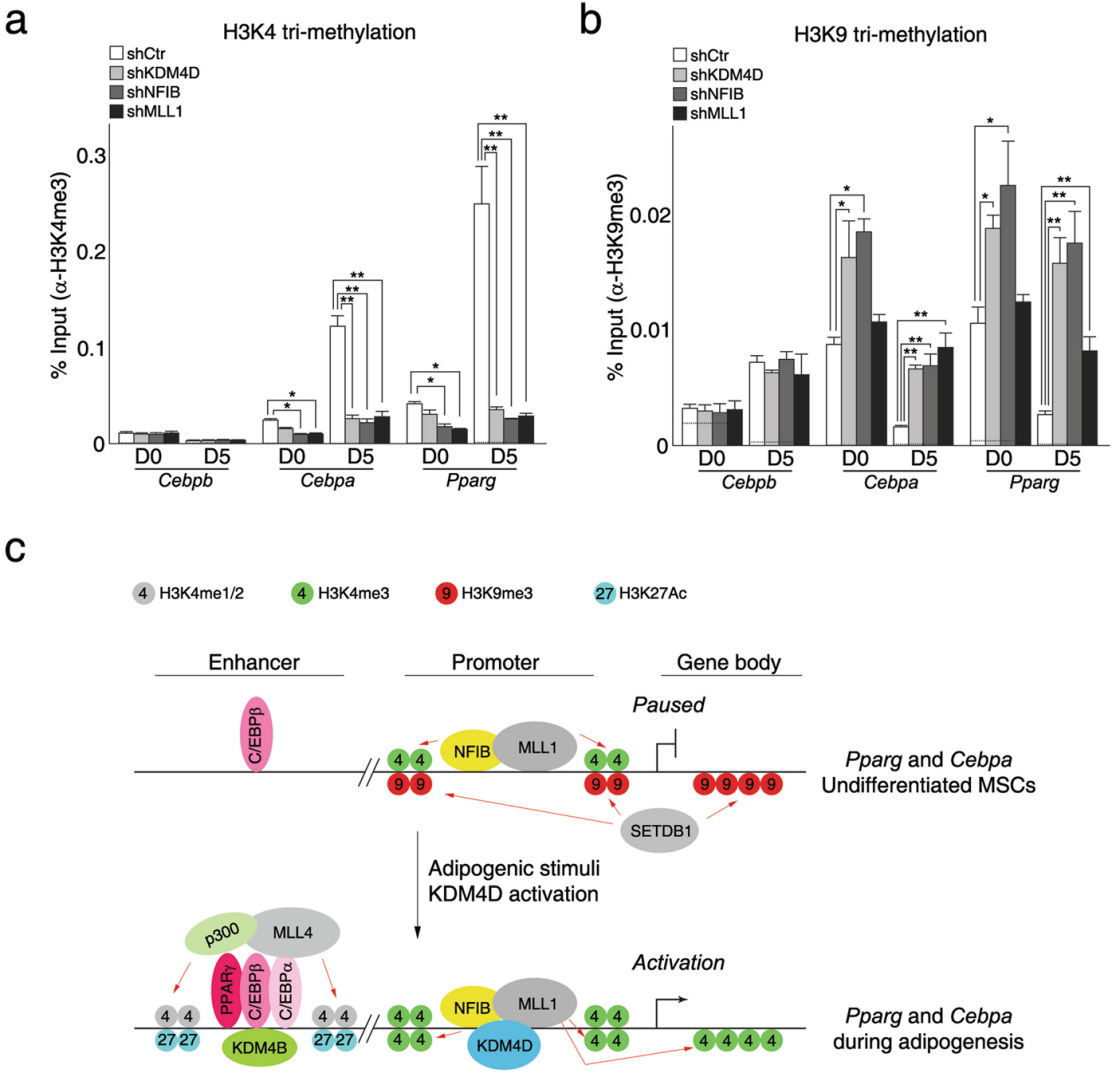
KDM4D coordinates H3K4me3/H3K9me3 to regulate the adipogenic differentiation of C3H10T1/2 mesenchymal stem cells. (**a,b**) KDM4D is required for the tri-methylation of H3K4 as well as the demethylation of tri-methylated H3K9 at the *Cebpa* and *Pparg* promoters in adipogenic differentiation. Chromatins prepared from control cells and three KD cells (shKDM4D, shNFIB, and shMLL1) before (day 0) and after (day 5) the induction of differentiation were precipitated with IgG (**a,b**), α-H3K4me3 (**a**), and α-H3K9me3 (**b**) antibodies. The data are presented as the % input of indicated antibodies and the dotted lines indicate the % input of IgG. Quantitative PCR data in all figures are representative of three independent experiments and are presented as mean ± SD. *p < 0.05; **p < 0.01. (**c**) A model for the roles of KDM4D, NFIB, and MLL1 complex in the transcription activation of *Cebpa* and *Pparg* during adipogenic differentiation.

Finally, we examined whether KDM4D modulates the H3K4me3/H3K9me3 dynamics during adipogenic differentiation. Chromatin immunoprecipitation assays showed that the depletion of NFIB, MLL1, or Ash2l led to significant decreases in the levels of H3K4me3 at the promoters of *Pparg* and *Cebpa* (Fig. 6a and Supplementary Fig. S5d). In particular, reduced levels of H3K4me3 at the *Pparg* and *Cebpa* promoters were observed, even before adipogenic induction by MDI in both shNFIB and shMLL1 cells, suggesting that NFIB and MLL1 complex might contribute to the tri-methylation of H3K4 at those promoters in both undifferentiated and differentiated C3H10T1/2 cells. In contrast, no significant change in H3K4me3 was observed at the *Cebpb* promoter in those cells tested (Fig. 6a), which is consistent with previous data showing that KDM4D is not related directly to the transcription activation of *Cebpb*. Surprisingly, despite the KDM4D-independent bindings of NFIB and MLL1 complex to the target promoters (Fig. 5g–i), significantly lower levels of H3K4me3 were detected after MDI induction - in cells depleted of KDM4D than in the control cells (Fig. 6a). Moreover, the levels of H3K9me3, which were decreased dramatically at both *Pparg* and *Cebpa* promoters during adipogenic differentiation (Supplementary Fig. S4h), were similar in the tested KD cells to those observed in undifferentiated control cells (Fig. 6b). These results strongly suggest that the demethylation of H3K9me3 by KDM4D should precede for MLL1 complex to tri-methylate H3K4 and activate the transcription of *Pparg* and *Cebpa*, which should be sufficient to drive adipogenic differentiation in C3H10T1/2 mesenchymal stem cells.

## Conclusions

In summary, our findings that KDM4D is required for the adipogenic differentiation of C3H10T1/2 MSCs suggests KDM4D as a critical regulator of adipogenesis. In addition, the physical interaction of KDM4D with NFIB and MLL1 complex, and their involvement in activating the transcription of key adipogenic regulators, such as *Pparg* and *Cebpa*, suggest the existence of regulatory complex comprised of KDM4D histone demethylase, NFIB transcription factor, and MLL1 histone lysine methyltransferase complex. Moreover, the coordinated bindings of KDM4D and its interacting partners to the *Pparg* and *Cebpa* promoters and resulting changes in the dynamics of H3K4me3/H3K9me3 provide a molecular mechanism through which a sequence-specific transcription factor and histone modifying enzymes cooperate to promote the adipogenic differentiation in C3H10T1/2 MSCs (Fig. 6c). In our proposed model, we speculate that NFIB, MLL1 complex and adipogenic pioneer transcription factor C/EBPβ bind to the promoter and enhancer of *Pparg* and *Cebpa* in undifferentiated MSCs. Although NFIB and MLL1 complex mediates the tri-methylation of H3K4 at the promoter of *Pparg* and *Cebpa* to some extent, their function to activate transcription is counteracted by the repressive histone mark, H3K9me3, established by SETDB1. Hence, these key adipogenic regulators remain in the primed but paused state. Upon adipogenic stimuli, an increased level of KDM4D and its recruitment to the target promoters through an interaction with NFIB and MLL1 complex leads to the removal of repressive H3K9me3 marks, which allows the cells to escape from a ‘primed but paused’ state. Finally, coordinated actions by KDM4D, NFIB, and MLL1 complex as well as enhancer activation modulated by MLL4 complex drive the cells to differentiate into adipocytes. In conclusion, our study strongly supports the idea of regulatory complex comprised of KDM4D, NFIB, and MLL1 complex being the critical regulator of adipogenic differentiation in C3H10T1/2 MSCs. Nevertheless, it is unclear if they are also involved in differentiation to other lineages, which needs to be determined in further studies.

## Methods

### Plasmid DNAs

The full-length cDNAs used in this study were either amplified directly from mouse cDNA (*Kdm4d*) or purchased from Origene (*Nfib*: MR206682, Rockville, MD, USA), Addgene (*Kdm4d*: #61553, *Cebpa*: #66978, *Cebpb*: #66979, *Wdr5*: #15552, Watertown, MA, USA), and Korea Human Gene Bank (*Kdm4a*: mMU005272, *Kdm4c*: mMU003529, *Pparg*: mMU001014, Daejeon, Korea). The construction of FLAG- or HA- epitope tagged genes was carried out using the standard protocols by PCR. Complete sequences of all PCR-amplified products were verified by sequencing. The detailed information regarding the plasmids and primer sets used to construct the epitope-tagged genes is provided in Supplementary Table S1.

### Cell cultures and *in vitro* differentiation

Mouse C3H10T1/2 mesenchymal stem cells and human 293 T cells were maintained in Dulbecco’s modified Eagle’s medium (DMEM, WELGENE, Gyeongsan, Korea) supplemented with 10% fetal bovine serum (FBS, WELGENE, Gyeongsan, Korea) and antibiotics in a humidified atmosphere with 5% CO2 at 37 °C. Adipogenesis was induced by treatment of two day post confluent C3H10T1/2 MSCs (designated by day 0 or D0) with 10  µg/ml insulin (Sigma, St Louis, MO, USA), 1  µM dexamethasone (Sigma, St Louis, MO, USA), and 0.5  mM isobutylmethylxanthine (Sigma, St Louis, MO, USA) in the presence of 10% FBS until day two. The cells were then fed with DMEM supplemented with 10% FBS and 10  µg/ml insulin for two days, after which they were fed every other day with DMEM containing 10% FBS. Differentiation was monitored initially by the appearance of lipid droplets in the cells and then confirmed by Oil Red O (ORO) staining. For ORO staining, the cells were washed three times with PBS (GIBCO, Grand Island, NY, USA) and fixed for 1  h with 10% formaldehyde (Sigma, St Louis, MO, USA). Subsequently, 0.35% Oil red O (Sigma, St Louis, MO, USA) in isopropanol was diluted with water (3:2), filtered through a 0.22  µm filter, and added to the fixed cells for 20  min at room temperature. The cells were then washed with water, and stained lipid droplets were visualized using an Olympus BX50 optical microscope. All images were taken using a 30× objective and processed using the Adobe Photoshop CC 2015 software.

### Transfection and retroviral infection

The cells were transfected using polyethyleneimine (PEI, Sigma, St Louis, MO, USA) or WelfectEX^TM^ PLUS Reagent (WELGENE, Gyeongsan, Korea) according to the manufacturer’s recommendations. For a retroviral infection, replication-defective retroviruses were made in ecotropic phoenix retroviral packaging cells (ATCC, Manassas, VA, USA) by transfection of the relevant retroviral vectors using the WelfectEXTM PLUS Reagent. The transfected cells were selected with 5  µg/ml of puromycin (Sigma, St Louis, MO, USA) for 24  h and the cells were then replenished with fresh medium without puromycin. Twenty-four hours after replacing with the medium, the viral supernatants were collected and filtered through a 0.45  µm filters. C3H10T1/2 MSCs were seeded one day before infection, and infected with the viral supernatants for two consecutive days. To enhance the infection efficiency, 8  µg/ml of polybrene (Sigma, St Louis, MO, USA) was added to the viral supernatants.

### Establishment of stable cell lines expressing shRNA

C3H10T1/2 MSCs were transfected with pLKO.1 containing shRNA against mouse *Kdm4d*, *Nfib*, *Mll1*, or *Ash2l* and then selected in the presence of puromycin (5  µg/ml) for two weeks. Puromycin-resistant clones were isolated and tested for knockdown efficiency by RT-qPCR. For each target, at least two independent clones were selected, and the expression of targeted genes was confirmed by immunoblot analysis before being used for further study. Sequence information regarding the shRNAs used in this study is provided in the supplemental information (Supplementary Table S2) and can also be found at http://www.broadinstitute.org/rnai/public/clone/search.

### Immunoblot and co-immunoprecipitation assay

The whole cell extracts were prepared by lysing the cell pellets on ice for 10  min with RIPA buffer (10  mM Tris-HCl, pH 7.5, 1  mM EDTA, pH 8.0, 150  mM NaCl, 1% Triton X-100, 1% sodium deoxycholate, and 0.1% SDS) in the presence of a protease inhibitor cocktail (Roche, Mannheim, Germany) followed by 20  s of sonication using a VCX130 (Sonics, Newtown, CT, USA) sonicator (power setting 20% amplitude), and centrifugation at 12,000 × g for 20  min. For FLAG immunoprecipitation, proteins were incubated at 4 °C for 16  h in RIPA buffer-equilibrated FLAG-M2 agarose (Sigma, St Louis, MO, USA). For HA immunoprecipitation, proteins were precleared using protein A/G sepharose 4 Fast Flow (GE Healthcare, Madison, WI, USA) for 2  h, after which the precleared proteins were incubated at 4 °C for 16  h with anti-HA antibody followed by the immobilization of antibodies for 3  h with protein G sepharose 4 Fast Flow. Immuno-complexes were washed four times with RIPA buffer and eluted with SDS running buffer in the presence of β-mercaptoethanol (Merck, Darmstadt, Germany) for 5  min at 94 °C. The following antibodies were used for immunoblot assays: monoclonal anti-Flag M2 (Sigma, St Louis, MO, USA), monoclonal anti-Actin (Sigma, St Louis, MO, USA), monoclonal anti-HA (16B12) (Covance, Princeton, NJ, USA), monoclonal anti-PPARγ (Cell Signaling Technology, Boston, MA, USA), polyclonal anti-C/EBPα (Santa Cruz Biotechnology, Santa Cruz, CA, USA), polyclonal anti-aP2 (Abcam, Cambridge, UK), polyclonal anti-KDM4D (Abcam, Cambridge, UK), polyclonal anti-NFIB (Abcam, Cambridge, UK), polyclonal anti-Wdr5 (Abcam, Cambridge, UK), polyclonal anti-MLL1 (Bethyl Laboratories, Montgomery, TX, USA), polyclonal anti-Ash2l (Bethyl Laboratories, Montgomery, TX, USA), polyclonal anti-Menin (Bethyl Laboratories, Montgomery, TX, USA), polyclonal anti-PTIP (Bethyl Laboratories, Montgomery, TX, USA), and polyclonal anti-Rbbp5 (Bethyl Laboratories, Montgomery, TX, USA). Detailed information regarding the antibodies, including working concentrations, is provided in the supplementary information (Supplementary Table S3).

### RT-qPCR

For quantitative reverse transcription PCR (RT-qPCR) analysis, the total RNA was isolated using an RNeasy plus mini kit (Qiagen, Hilden, Germany) according to the manufacturer’s instructions. cDNA synthesis was then carried out using a GoScript reverse transcription system (Promega, Madison, WI, USA) as recommended by the manufacturer. qPCR was conducted using an ABI 7300 Real-Time PCR system (Applied Biosystems, Foster City, CA, USA) with SYBR Green I (Invitrogen, Carlsbad, CA, USA) and i-StarTaq DNA polymerase (Intron, Sungnam, Korea). The relative mRNA level was first quantified using the standard curve method, after which the data were normalized to GAPDH mRNA. The primer sets used in this study can be found in the supplementary information (Supplementary Table S4).

### ChIP-qPCR

For the chromatin immunoprecipitation assays, 100–300  µg of sonicated chromatins were precleared for 2  h using protein A/G sepharose 4 Fast Flow in the presence of 4  mg/ml salmon sperm DNA and 0.5  mg/ml bovine serum albumin, followed by immunoprecipitation using the relevant antibodies. The purified DNA was analyzed by quantitative PCR (qPCR) on an ABI 7300 Real-Time PCR system with SYBR Green I and iStarTaq DNA polymerase. For quantification, the % input value per sample was calculated using a standard curve method. The data were presented as % input or relative ChIP signal as indicated in the figures. The primers used for ChIP-qPCR analysis are listed in Supplementary Table S5.

### Liquid chromatography with tandem mass spectrometry (LC-MS/MS) analysis

Sample preparation for liquid chromatography with tandem mass spectrometry (LC-MS/MS) analysis was conducted as follows. C3H10T1/2 MSCs expressing full length FLAG-KDM4D were lysed with a hypotonic buffer (10  mM HEPES pH 7.9, 10  mM KCl, 0.1  mM EDTA pH 8.0, 0.3% NP-40, and protease inhibitor cocktail), then passed 10 times through a 26 G 1/2 needle. After incubating on ice for 10  min, the nuclei were harvested by centrifugation and the pellets were suspended with RIPA buffer followed by 150  s of sonication using a VCX130 sonicator (power setting 40% amplitude). The nuclear extracts were then incubated with the FLAG-M2 agarose for 16  h at 4 °C. The immunoprecipitated proteins were separated by 10% SDS-PAGE and visualized with Coomassie brilliant blue G-250. LC-MS/MS analysis, and protein identification using the MS/MS spectra were performed at the Korea Basic Science Institute (KBSI, Daejeon, Korea).

### RNA-Seq analysis

The total RNA was isolated from eight different samples (control MSCs, shKDM4D, shMLL1, and shNFIB cells at two time points) using an RNeasy plus mini kit according to the manufacturer’s instructions. The RNA quality was assessed with an Agilent Bioanalyzer-2100 using the RNA 6000 Nano Chip (Agilent Technologies, Santa Clara, CA, USA) and RNA quantification was performed using an ND-2000 Spectrophotometer (Thermo Fisher Scientific, Waltham, MA, USA). Library preparation with SMARTer Stranded RNA-Seq Kit (Clontech Laboratories, Palo Alto, CA, USA), HiSeq 2500 sequencing, and raw data generation were conducted by eBiogen (Seoul, Korea). The high-quality sequence reads from each sample were assembled and mapped to the annotated mouse genomic DNA (Genome Reference Consortium Mouse Build 38; GRCm38/mm10) using a CLC Genomic Workbench 11.0.1 (Qiagen, Hilden, Germany) with the following parameters: mismatch cost 2, insertion cost 3, deletion cost 3, length fraction 0.8, and similarity fraction 0.8. Only protein coding genes with RPKM (Reads Per Kilobase of exon model per Million mapped reads) >1.0 were regarded as expressed and the genes with RPKM > 1.0 in at least one sample were kept for subsequent analysis. For differentially expressed gene (DEG) analysis, the total read counts were normalized in quantiles by RPKM before calculating gene expression levels and fold changes (FC). For cluster analysis, the genes with expression changes during adipogenic differentiation of C3H10T1/2 MSCs (between days 0 and 5) that were >2-fold, and FDR < 0.05 were selected for further analysis. The expression changes in these selected genes in shKDM4D, shMLL1, and shNFIB cells were calculated and subjected to cluster analysis using hclust within the R package. A heat map was generated by Pheatmap within the R package. Gene ontology (GO) analysis and KEGG pathway analysis were conducted using DAVID bioinformatics resources 6.8.

### Statistical analysis

The data shown for all qPCR-based experiments are representative of at least three independent experiments as indicated in the figure legends and are presented as the means ± SD. Statistical significance and *p* values were determined by Student’s t-tests of the indicated paired groups.

### Data resources

The RNA-seq data were deposited to the Gene Expression Omnibus (GEO) repository (NCBI) under the accession number: GSE131369.

## Supplementary information

Supplementary information.

Dataset 1.

Dataset 2.

Dataset 3.

## Data Availability

The data generated and analyzed during this study are available from the corresponding author on request.
