# Supplementary material for: Histone demethylase KDM4D cooperates with NFIB and MLL1 complex to regulate adipogenic differentiation of C3H10T1/2 mesenchymal stem cells
Source: Sci Rep. 2020 Feb 20;10:3050. doi: 10.1038/s41598-020-60049-8 (PMC7033117; doi:10.1038/s41598-020-60049-8)
Supplement: Supplementary file 1 — Supplementary information. [file 41598_2020_60049_MOESM1_ESM.pdf]

Supplementary Information

Histone demethylase KDM4D cooperates with NFIB and MLL1 complex to regulate adipogenic differentiation of C3H10T1/2 mesenchymal stem cells

Jang Hyun Choi and Hansol Lee*

Department of Biological Sciences, College of Natural Science, Inha University,

100 Inha-ro, Michuhol-gu, Incheon, Korea, 22212

* Address correspondence to Hansol Lee, hlee@inha.ac.kr

List of contents:

- Supplementary Figure legends (pages 2 to 5)
- Supplementary Figure S1 ~ S5 (pages 6 to 10)
- Supplementary Table S1 ~ S5 (pages 11 to 16)
- Uncropped blots for Figures (pages 17 to 27)
- References (Pages 28 to 29)

Supplementary Figure legends

Supplementary Figure S1. KDM4D is required for adipogenic differentiation of C3H10T1/2 mesenchymal stem cells. (a and b) Expression of KDM4D increases during adipogenic differentiation. Cells were collected before (day 0) and after the induction of differentiation (day 1, 5, and 8). (a) RT-qPCR analysis of the KDM4D family of genes (*Kdm4a* ~ *Kdm4d*) as well as key adipogenic marker genes (*Cebpb*, *Pparg*, *Cebpa*, and *aP2*) during adipogenic differentiation of C3H10T1/2 cells. The total RNAs were isolated from the cells at indicated time points and the relative mRNA levels were measured by RT-qPCR. (b) Immunoblot analysis of KDM4D, PPARγ, C/EBPα, and aP2 during adipogenic differentiation. Actin was used as a control. (c) Establishment of stable cell lines depleted of endogenous KDM4D (shKDM4D-1 and shKDM4D-2). RT-qPCR analysis of *Kdm4d* and immunoblot analysis of endogenous KDM4D (inset) in the control cells, shKDM4D-1, and shKDM4D-2 cells. (d) Exogenous expression of FLAG-KDM4D lacking 3'-UTR rescues adipogenesis in shKDM4D-1 cells. The total RNAs were isolated from the control cells, shKDM4D-1, and shKDM4D-1 infected with the retrovirus expressing FLAG-KDM4D, followed by RT-qPCR analysis of *Pparg* (left) and *aP2* (right). (e) Overexpression of KDM4D has little effects on adipogenic differentiation in C3H10T1/2 cells. C3H10T1/2 cells were infected with the retrovirus expressing empty vector or FLAG-KDM4D. The total RNAs were isolated from the cells at indicated time points and the mRNA levels of *Pparg* (left) and *aP2* (right) were quantified using RT-qPCR. In all figures including RT-qPCR analysis, the relative mRNA levels were presented as the ratio of mRNA level at each time point (or shKDM4D cells in Fig S1c) to the mRNA level in the control cells at day 0 following normalization to GAPDH. All qPCR data are representative of three independent experiments and are presented as mean ± SD. *$p < 0.05$; **$p < 0.01$.

Supplementary Figure S2. Depletion or overexpression of KDM4D has little effects on the expression of Wnt ligands in C3H10T1/2 cells. The total RNAs were prepared from the control,

shKDM4D-1, and control cells infected with the retrovirus expressing FLAG-KDM4D, followed by RT-qPCR analysis of Wnt ligands (*Wnt1*, *Wnt6*, *Wnt10a*, and *Wnt10b*). The relative mRNA levels were presented as a ratio to the mRNA level in the control cells following normalization to GAPDH. qPCR data are representative of three independent experiments and are presented as mean ± SD.

Supplementary Figure S3. **KDM4D interacts with NFIB transcription factor and MLL1 H3K4 methyltransferase complex.** (a) KDM4D interacts with NFIB in cells. Human 293T cells were transfected with pcDNA3 containing FLAG-NFIB, HA-KDM4D, or both as indicated. Protein extracts were immunoprecipitated with FLAG-M2 agarose and the interactions were confirmed by immunoblot analysis using α-HA antibody. (b and c) KDM4D and NFIB interact with MLL1 histone methyltransferase complex in human 293T cells. Cells were transfected with empty vector, pcDNA3 containing FLAG-KDM4D (b), or pcDNA3 containing FLAG-NFIB (c). The protein extracts were immunoprecipitated with FLAG-M2 agarose and the interactions were examined by immunoblot analysis using indicated antibodies. Actin was used as a loading control. (d) JmjC domain of KDM4D mediates the interaction with Wdr5, a core subunit of MLL1 complex. Human 293T cells were co-transfected with plasmids containing full-length FLAG-Wdr5 and indicated HA-KDM4D (FL; full-length, numbered; deletion mutants). (e) N-terminal domain of NFIB is required for the interaction with Wdr5. Human 293T cells were co-transfected with plasmids containing full-length FLAG-Wdr5 and the indicated HA-NFIB (FL; full-length, numbered; deletion mutants). In (d) and (e), protein extracts were immunoprecipitated with α-HA antibody and the interactions were determined by immunoblot analysis using α-HA antibody.

Supplementary Figure S4. **KDM4D, NFIB, and MLL1 complex work together in regulating adipogenic differentiation.** (a – c) Establishment of stable cell lines depleted of (a) NFIB (shNFIB),

(b) MLL1 (shMLL1), and (c) Ash2l (shAsh2l). RT-qPCR analysis and immunoblot analysis (inset) of indicated target genes in the control and KD cells. Actin was used as a control. In (c), Oil Red O staining at day eight after the induction of differentiation shows defective adipogenic differentiation in cells depleted of Ash2l, a component of MLL1 complex. (d) RNA-seq heatmap depicting the changes in expression of the genes (downregulated during adipogenic differentiation of C3H10T1/2 cells) in the control, shKDM4D, shNFIB, and shMLL1 cells. The color intensity scale was included at the bottom of the heatmap. The threshold for down-regulation is 2.0 fold. (e) Schematic diagram of the identification of KDM4D-, NFIB-, and MLL1-dependent genes (left) and Venn diagram of the genes affected by KDM4D, NFIB, and MLL1 depletion (higher than the control cells at day 5). (f) Ash2l binds to the promoter of *Cebpa*, and *Pparg* genes. Chromatins prepared from C3H10T1/2 cells after (day 5) the induction of differentiation were precipitated with IgG or α-Ash2l antibody, followed by quantitative PCR analysis. For the relative ChIP signal, the % input was calculated for each sample and data are presented as a ratio of the % input (α-Ash2l) to the % input (IgG). (g and h) Dynamics of histone modifications (H3K4me3 in G and H3K9me3 in H) before (day 0) and after (day 5) the induction of differentiation in C3H10T1/2 cells. Chromatins prepared from the cell at indicated time points were precipitated with α-H3K4me3 (g) and α-H3K9me3 (h) antibodies. Data are presented as the % input of indicated antibodies. Quantitative PCR data in all figures are representative of three independent experiments and are presented as mean ± SD. **p* < 0.05; ***p* < 0.01.

Supplementary Figure S5. **Ash2l, a component of MLL1 complex, is required for the adipogenic differentiation in C3H10T1/2 mesenchymal stem cells.** (a – c) Ash2l is required for the interaction between KDM4D and NFIB and their bindings to the *Pparg* and *Cebpa* promoters. Control and shAsh2l cells were infected with a retrovirus expressing empty vector or indicated FLAG-tagged proteins. The total protein extracts and chromatins were prepared from cells after the induction of differentiation (day 5), followed by immunoprecipitation assay and ChIP-qPCR analysis. (a) Immunoblot analysis of the

interaction between KDM4D and NFIB in the control and shAsh2l cells. (b and c) ChIP-qPCR analysis of FLAG-NFIB (b) and FLAG-KDM4D (c) bindings to the promoters of *Cebpb*, *Cebpa*, and *Pparg* in the control and shAsh2l cells. For the relative ChIP signal, the % input was calculated for each sample and data are presented as a ratio of the % input in the cells expressing FLAG-NFIB (b) or FLAG-KDM4D (c) to the % input in the control cells infected with a retrovirus expressing empty vector. (d and e) Ash2l is required for the tri-methylation of H3K4 as well as the demethylation of H3K9me3 at the *Cebpa* and *Pparg* promoters in adipogenic differentiation. Chromatins prepared from the control and shAsh2l cells before (day 0) and after (day 5) the induction of differentiation were precipitated with IgG (d and e), α-H3K4me3 (d), and α-H3K9me3 (e) antibodies. Data are presented as the % input. Quantitative PCR data in all figures are representative of three independent experiments and are presented as mean ± SD. *$p < 0.05$; **$p < 0.01$.

Supplementary Figure S1

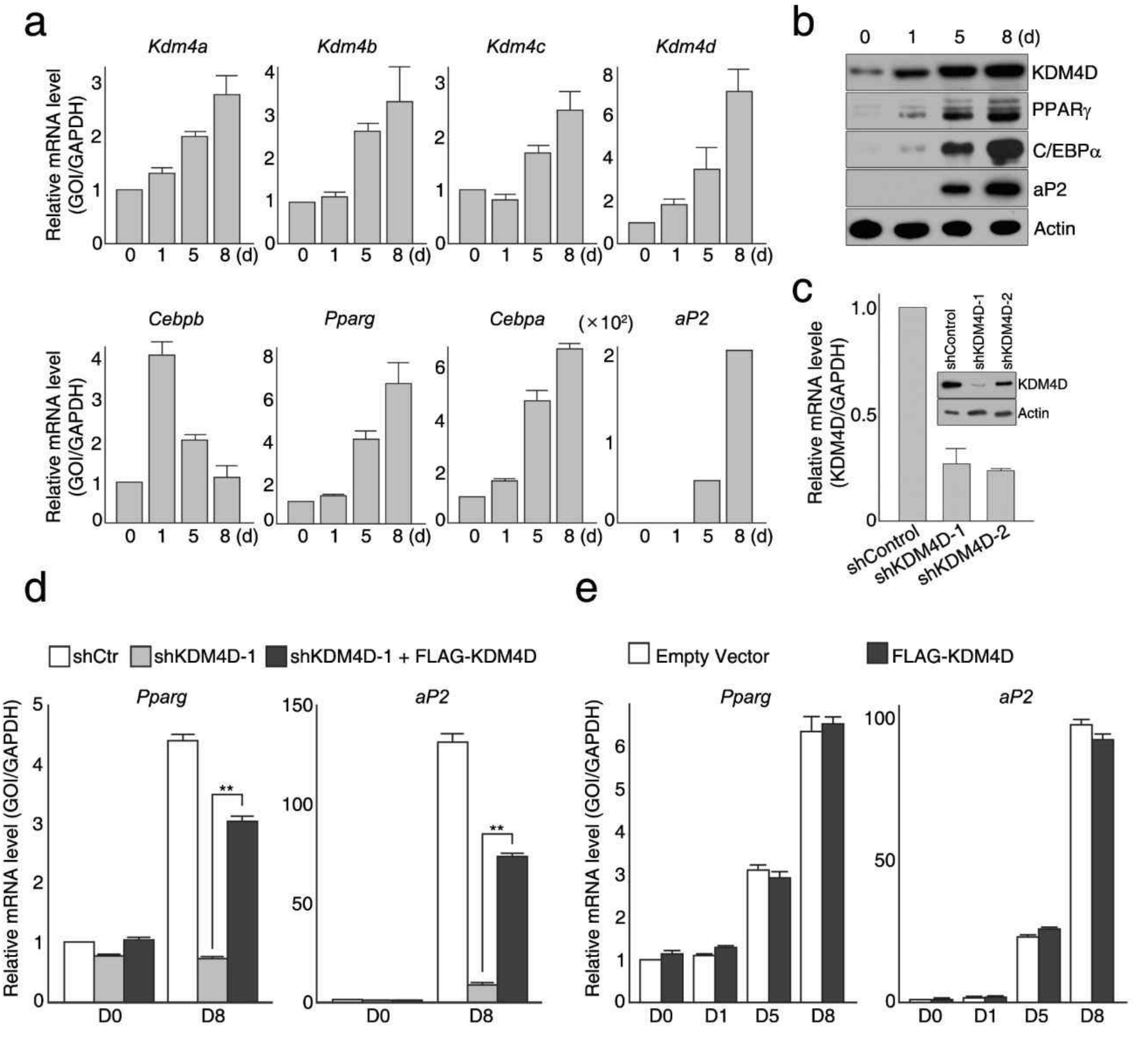

Supplementary Figure S2

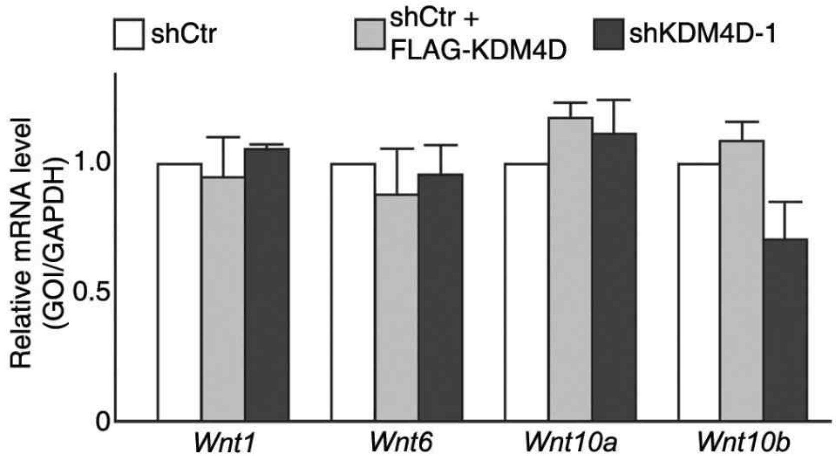

Supplementary Figure S3

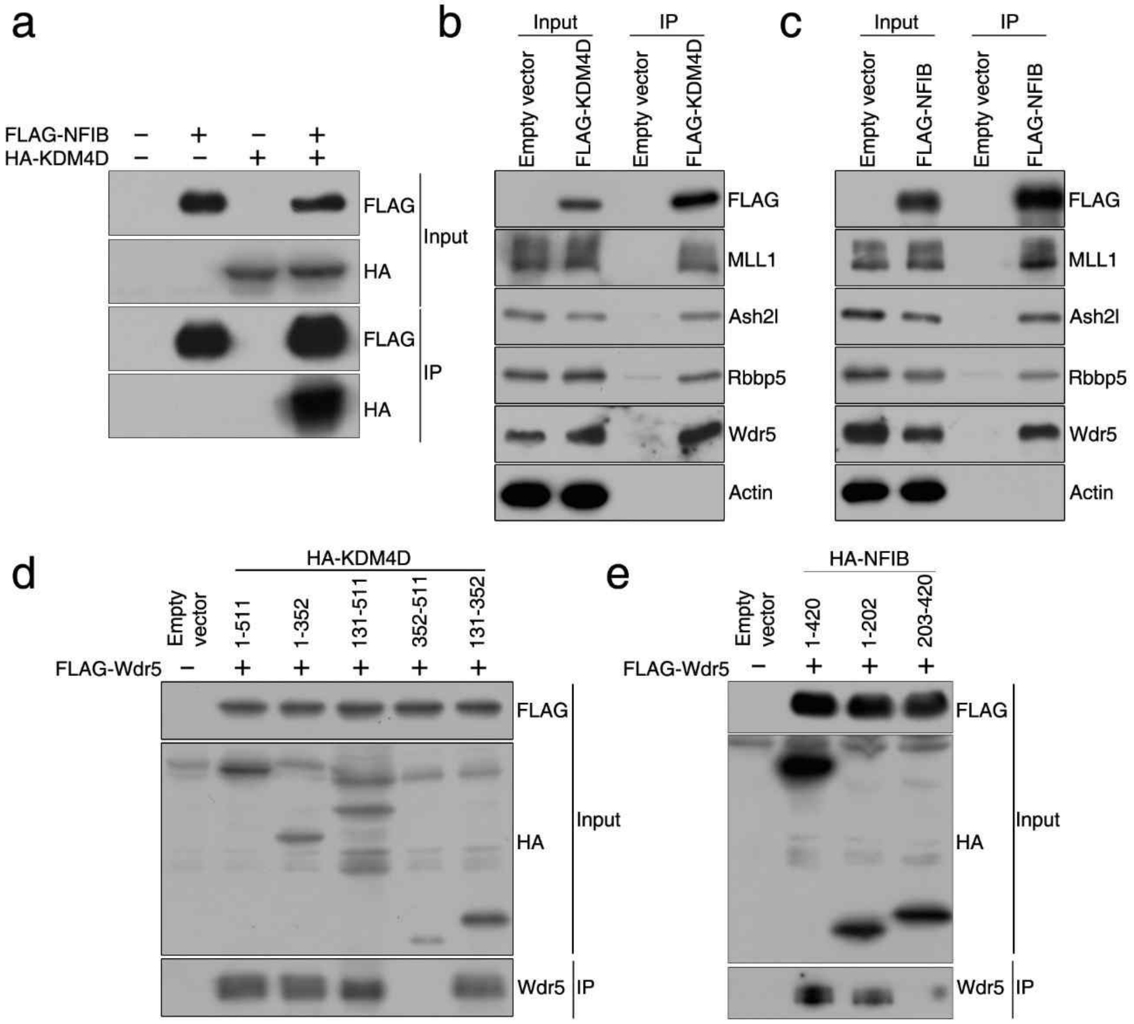

Supplementary Figure S4

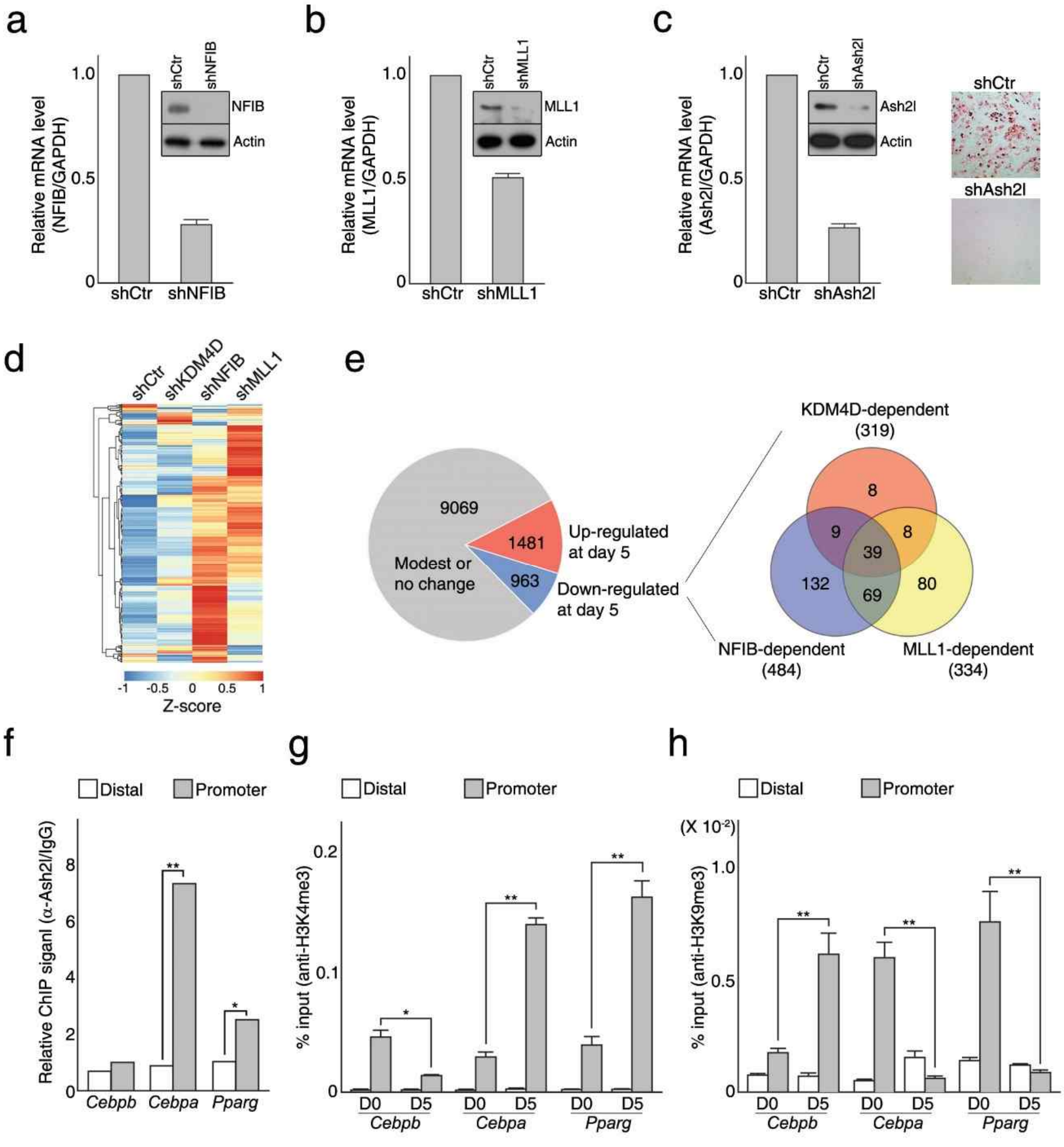

Supplementary Figure S5

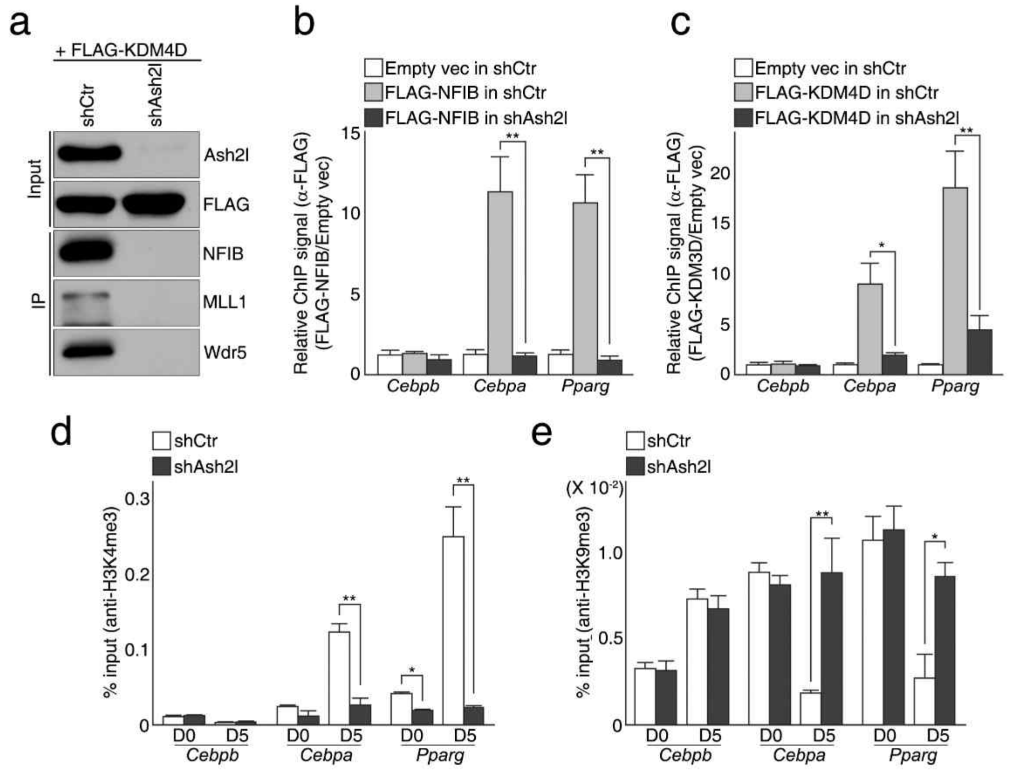

Supplementary Table S1. **Information of the plasmids used in this study**

Name	Source	Identifier	Primer	Cloning vector
FLAG-KDM4A	Korea Human Gene Bank (KHGB)	mMU005272	F: GATCGGATCCACCATGGATTACAAGGATGACGACGATAAGGTGGCTTCTGAATCAGAA<br>R: GATCCTCGAGCTAGATGCTATTAGAAAT	pLZRS (Dr. Nolan GP.)
FLAG-KDM4B	[1]	N/A		
FLAG-KDM4C	Korea Human Gene Bank (KHGB)	mMU003529	F: GATCGGATCCACCATGGATTACAAGGATGACGACGATAAGGTGGAGGTGGTGGAGGTG<br>R: GATCCTCGAGCTACTGTCTCTTCTGACA	pLZRS-IRES-GFP (Dr. Nolan GP.)
KDM4D	This study	N/A	F: GTACGTGAAGACGAAGTCCA<br>R: GTACTTAGGGGTCGGAGGT	pBluescript KS (+) (Invitrogen)
FLAG-KDM4D	Addgene	#61553	N/A	pLZRS
FLAG-KDM4D	This study	N/A	F: GATCGGATCCACCATGGATTACAAGGATGACGACGATAAGGTGAAGACGAAGTCCACATG<br>R: GATCCTCGAGTTAGGGGTCGGAGGTCAA	pcDNA3 (Invitrogen) and pLZRS-IRES-GFP
FLAG-KDM4D	This study	N/A	F: GATCGGATCCACCATGGATTACAAGGATGACGACGATAAGGTGAAGACGAAGTCCACATG<br>R: GATCCTCGAGTTAGGGGTCGGAGGTCAA	pMSCVpuro (Clonetech)
HA-KDM4D	This study	N/A	F: GATCGGATCCACCATGTACCCATACGATGTTCCAGATTACGCTGTGAAGACGAAGTCCACATG<br>R: GATCCTCGAGTTAGGGGTCGGAGGTCAA	pcDNA3
HA-KDM4D (1-352)	This study	N/A	F: GATCGGATCCACCATGTACCCATACGATGTTCCAGATTACGCTGTGAAGACGAAGTCCACATG<br>R: GATCCTCGAGTTAACTGGTAGACACCATAGT	pcDNA3
HA-KDM4D (131-511)	This study	N/A	F: GATCGGATCCACCATGTACCCATACGATGTTCCAGATTACGCTATTTATGGTGCTGACGTC<br>R: GATCCTCGAGTTAGGGGTCGGAGGTCAA	pcDNA3

HA-KDM4D (352-511)	This study	N/A	F: GATCGGATCCACCATGTACCCATACGATGTTCCAGATTACGCTCA GGAGCTCACCACCCGG<br>R: GATCCTCGAGTTAGGGGTCGGAGGTCAA	pcDNA3
HA-KDM4D (131-352)	This study	N/A	F: GATCGGATCCACCATGTACCCATACGATGTTCCAGATTACGCTAT TTATGGTGCTGACGTC<br>R: GATCCTCGAGTTAACTGGTAGACACCATAGT	pcDNA3
FLAG-NFIB	OriGene	MR206682	F: GATCGGATCCACCATGGATTACAAGGATGACGACGATAAGGTAA TGTATTCTCCCATCTGT<br>R: GATCGTCGACCTAGCCCAGGTACCAGGACTG	pcDNA3 and pLZRS-IRES-GFP
FLAG-NFIB	This study	N/A	F: GATCGGATCCACCATGGATTACAAGGATGACGACGATAAGGTAA TGTATTCTCCCATCTGT<br>R: GATCGTCGACCTAGCCCAGGTACCAGGACTG	pMSCVpuro
HA-NFIB	This study	N/A	F: GATCGGATCCACCATGTACCCATACGATGTTCCAGATTACGCTGT AATGTATTCTCCCATCTGT<br>R: GATCGTCGACCTAGCCCAGGTACCAGGACTG	pcDNA3
HA-NFIB (1-205)	This study	N/A	F: GATCGGATCCACCATGTACCCATACGATGTTCCAGATTACGCTGT AATGTATTCTCCCATCTGT<br>R: GATCGTCGACCTACTTGGCAGGATCACT	pcDNA3
HA-NFIB (206-420)	This study	N/A	F: GATCGGATCCACCATGTACCCATACGATGTTCCAGATTACGCTAA TCCTCCAGGGTACCTC<br>R: GATCGAATTCCTAGCCCAGGTACCAGGACTG	pcDNA3
FLAG-C/EBPα	Addgene	#66978	N/A	pLZRS
FLAG-C/EBPβ	Addgene	#66979	N/A	pLZRS
FLAG-PPARγ	Korea Human Gene Bank (KHGB)	mMU001014	F: GATCCTCGAGACCATGGATTACAAGGATGACGACGATAAGGTGG GTGAAACTCTGGGA<br>R: GATCCTCGAGCTAATACAAGTCCTTGTAGATCTCCTG	pcDNA3 and pLZRS-IRES-GFP
FLAG-Wdr5	Addgene	#15552	N/A	

Supplementary Table S2. Information of shRNAs used in this study

Name	Clone ID	Target sequence
shKDM4D-1	TRCN0000103540	CCCTAAGTCCATTACCTCATA
shKDM4D-2	TRCN0000103542	CCACGGTAAGTAACGTTCCTT
shNFIB-1	TRCN0000012088	CCACAACCATAGTATAAGAAA
shNFIB-2	TRCN0000012091	CCTGTTCAAAGGCATCCCTTT
shMLL1-1	TRCN0000034424	CGCGGTATTATCCTAATTTAA
shMLL1-2	TRCN0000034426	CGCCTTCACTTGACCATAATT
shAsh2l-1	TRCN0000034399	CCTGTGTCTGTGTGTTCCAAA
shAsh2l-2	TRCN0000034400	CCATTTAACAAAGATGGCTAT

Supplementary Table S3. Information of antibodies used in this study

Antibodies	Source	Identifier	Lot#	Application
KDM4D	Abcam	ab93694	GR280734-2	WB (1/2,000)
NFIB	Abcam	ab186738	GR229339-9	WB (1/2,000)
MLL1	Bethyl	A300-086A	6	WB (1/10,000)<br>ChIP (4ug)
MLL3	Dr. J. H. Kim	N/A	N/A	WB (1/1,000)
Ash2l	Bethyl	A300-489A	2	WB (1/10,000)<br>ChIP (4ug)
Actin (AC-15)	Sigma	A1987	N/A	WB (1/50,000)
aP2	Abcam	ab92501	GR106922-2	WB (1/3,000)
C/EBPα (14AA)	SantaCruz	sc-61	F3016	WB (1/1,000)
FLAG M2	Sigma	F1804	N/A	WB (1/3,000)
H3K4me3	Abcam	ab8580	GR13175719-3	ChIP (4ug)
H3K9me3	Abcam	ab8898	GR131093-2	ChIP (4ug)
HA (16B12)	Covance	MMS-101R	B224726	WB (1/1,000)<br>IP (1/200)
Menin	Bethyl	A300-105A	8	WB (1/5,000)
PPARγ (81B8)	Cell signaling	#2443	4	WB (1/1,000)
PTIP	Bethyl	A300-370A	1	WB (1/5,000)
Rabbit IgG	Vectashield	I-1000	N/A	ChIP (4ug)
Rbbp5	Bethyl	A300-109A	N/A	WB (1/10,000)
Wdr5	Abcam	ab22515	GR191510-1	WB (1/3,000)

Supplementary Table S4. Information of primers for RT-qPCR used in this study

Name	Primer	Reference
Kdm4a	F: GAGGAAGACTGCTGCTTATGCTC<br>R: TCACATCCACTGGACTTCTTTCA	[1]
Kdm4b	F: TGCAGTCCCTGAGGTACGATT<br>R: CCCGACACTCTCTTCATACGC	[1]
Kdm4c	F: CCTTCAGCAGAGACACATTTCCT<br>R: TCCAAGATACTTTGCCCCATAGA	[1]
Kdm4d	F: CTGGAAGAATCGCCTGTATGAGT<br>R: GTCTTGAATTGTTCCCAGGTGAC	[1]
Nfib	F: CCGGAATACCTGGAGTCG<br>R: GAAATGGCAACGGTGAGG	[2]
Mll1	F: GGCTCCAGCAAGAACAAAAG<br>R: TCACACCTGCAAATGAGAGC	[3]
Ash2l	F: AGTCACTGCCGGACACCTACAAAG<br>R: TTTGTAGGTGTCCGGCAGTGA	[4]
aP2	F: TGCTGCAGCCTTTCTCACCT<br>R: AGCCCACTCCCACTTCTTTCA	[5]
Cebpa	F: CCCAGCGGTGCCTTGTGC<br>R: TCCTTCCCCCAGCCGTTAGTG	[5]
Cebpb	F: ACGACTTCCTCTCCGACCTCT<br>R: CGAGGCTCACGTAACCGTAGT	[6]
Pparg	F: GCCCTTTGGTGACTTTATGGA<br>R: GCAGCAGGTTGTCTTGGATG	[7]
Wnt1	F: CCTCCACGAACCTGTTGACG<br>R: GTTCTGTCGGATCAGTCGCC	[8]
Wnt6	F: GCGGAGACGATGTGGACTTC<br>R: ATGCACGGATATCTCCACGG	[9]
Wnt10a	F: CCACTCCGACCTGGTCTACTTTG<br>R: TGCTGCTCTTATTGCACAGGC	[9]
Wnt10b	F: ACCACGACATGGACTTCGGAGA<br>R: CCGCTTCAGGTTTTCCGTTACC	[8]
Gapdh	F: GCCTCTCTTGCTCAGTGTCC<br>R: TGCGACTTCAACAGCAACTC	[1]

Supplementary Table S5. Information of primers for ChIP used in this study

Name	Primer	Reference
Cebpa promoter	F: TGGAGACGCAATGAAAAAGA<br>R: CCACGGGCTCTTCAGAGTAG	[10]
Cebpa distal	F: TGGAGACGCAATGAAAAAGA<br>R: GGAGAAGGCATGCTCATGTT	[10]
Cebpb promoter	F: AGCAGGGTTGAGGGAACAG<br>R: CAGGGAGGGAAAGGGTAAGA	[11]
Cebpb distal	F: GCAACTTGTCAACACCCTGTCTCA<br>R: GCTTCCTGTCCCAACCTCATGTTT	[11]
Pparg promoter	F: CTGTACAGTTCACGCCCCTC<br>R: TCACACTGGTGTTTTGTCTATG	[12]
Pparg distal	F: CTTTTTAATTTAGAAGACACAGGTATTATC<br>R: TCAATATATCACTTGGTTCCCTTATTCCTG	[12]
Actin	F: TGGGAATGGGTCAGAAGGACTG<br>R: GGGTCATCTTTTCACGGTTGGC	[1]

Uncropped blots for Figures

Full-length/Uncropped blots used in the article are indicated according to each figure. Black squares represent the images used for figures in the article.

Fig. 1b

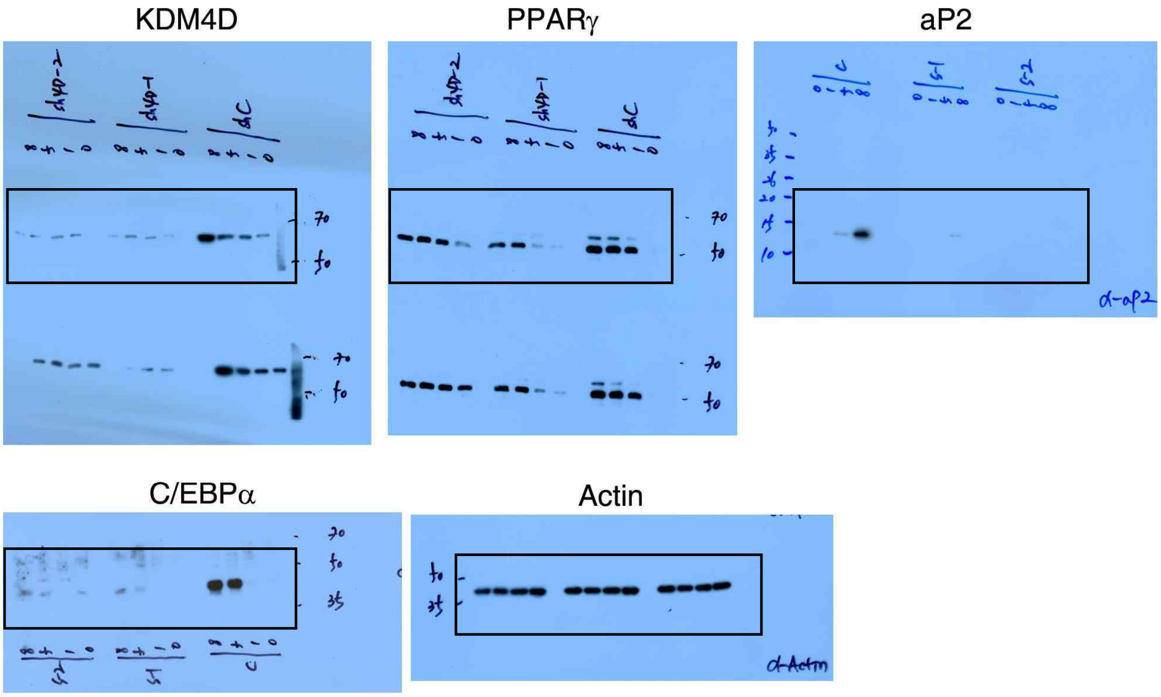

Fig. 1d

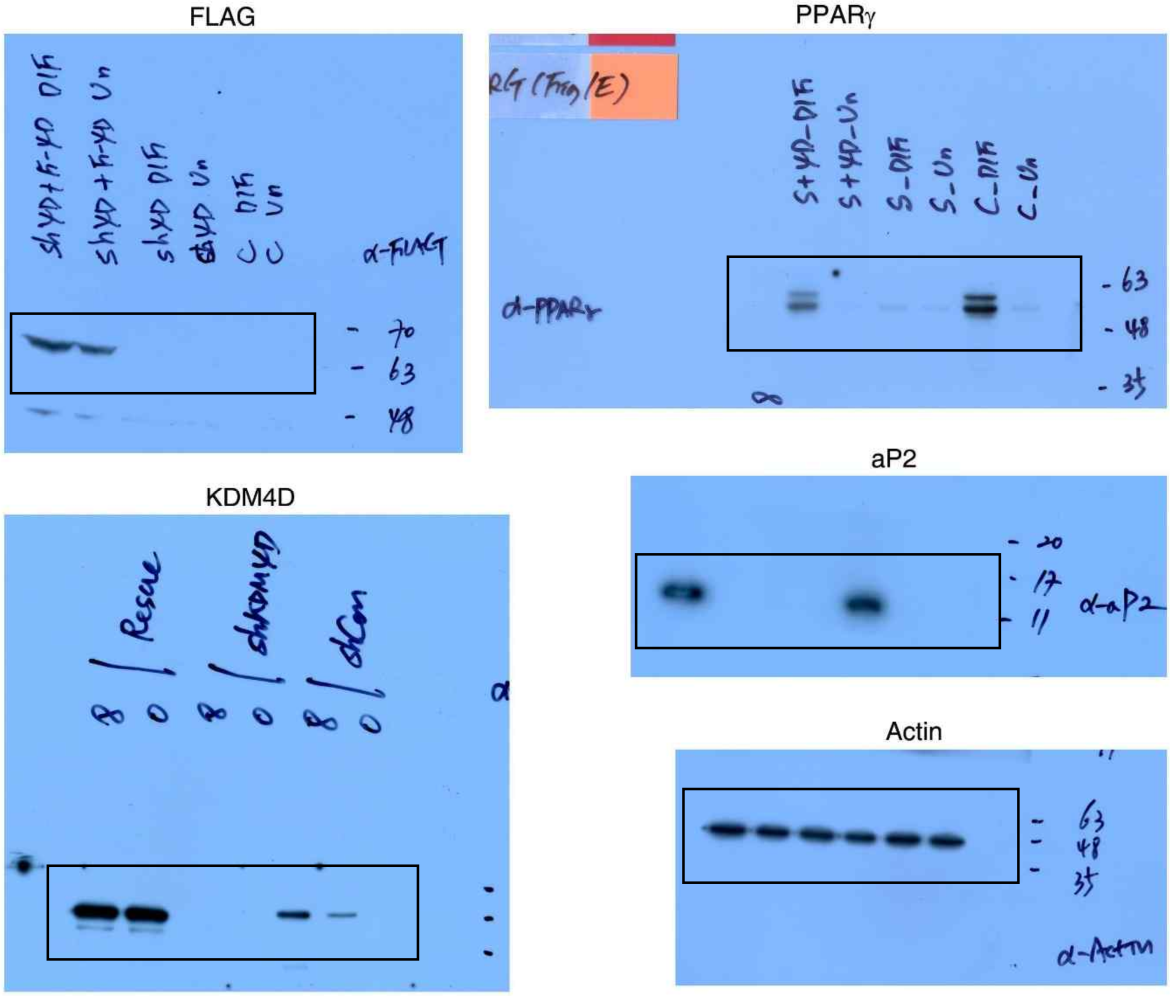

Fig. 1e

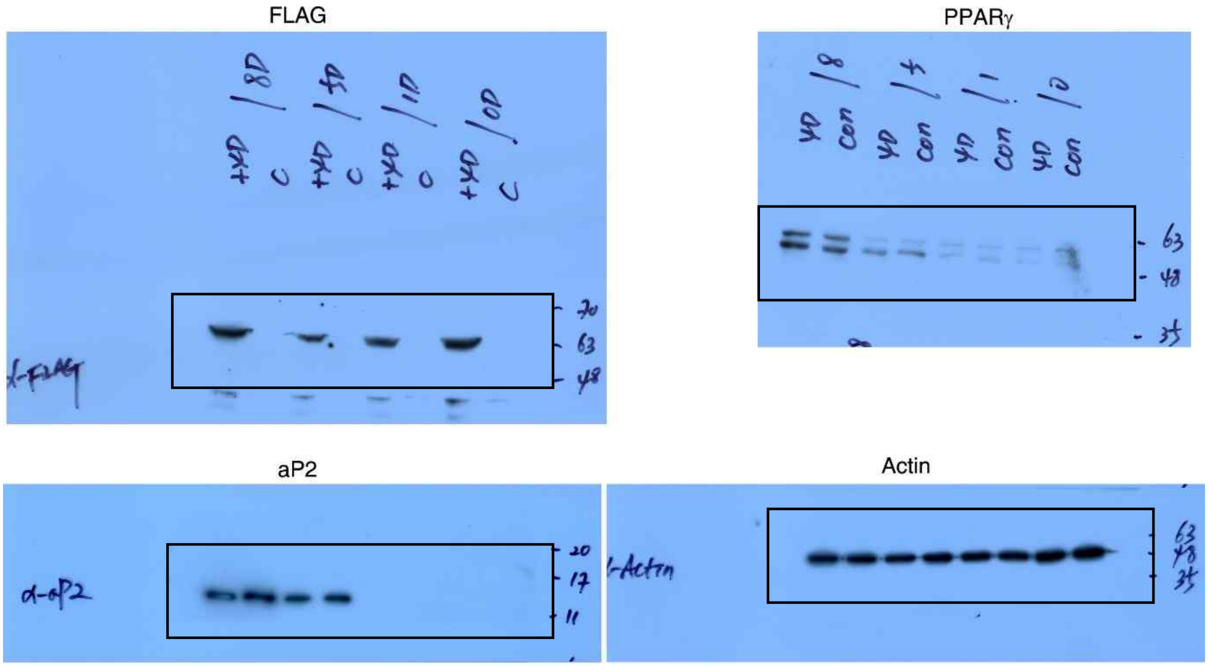

Fig. 2a

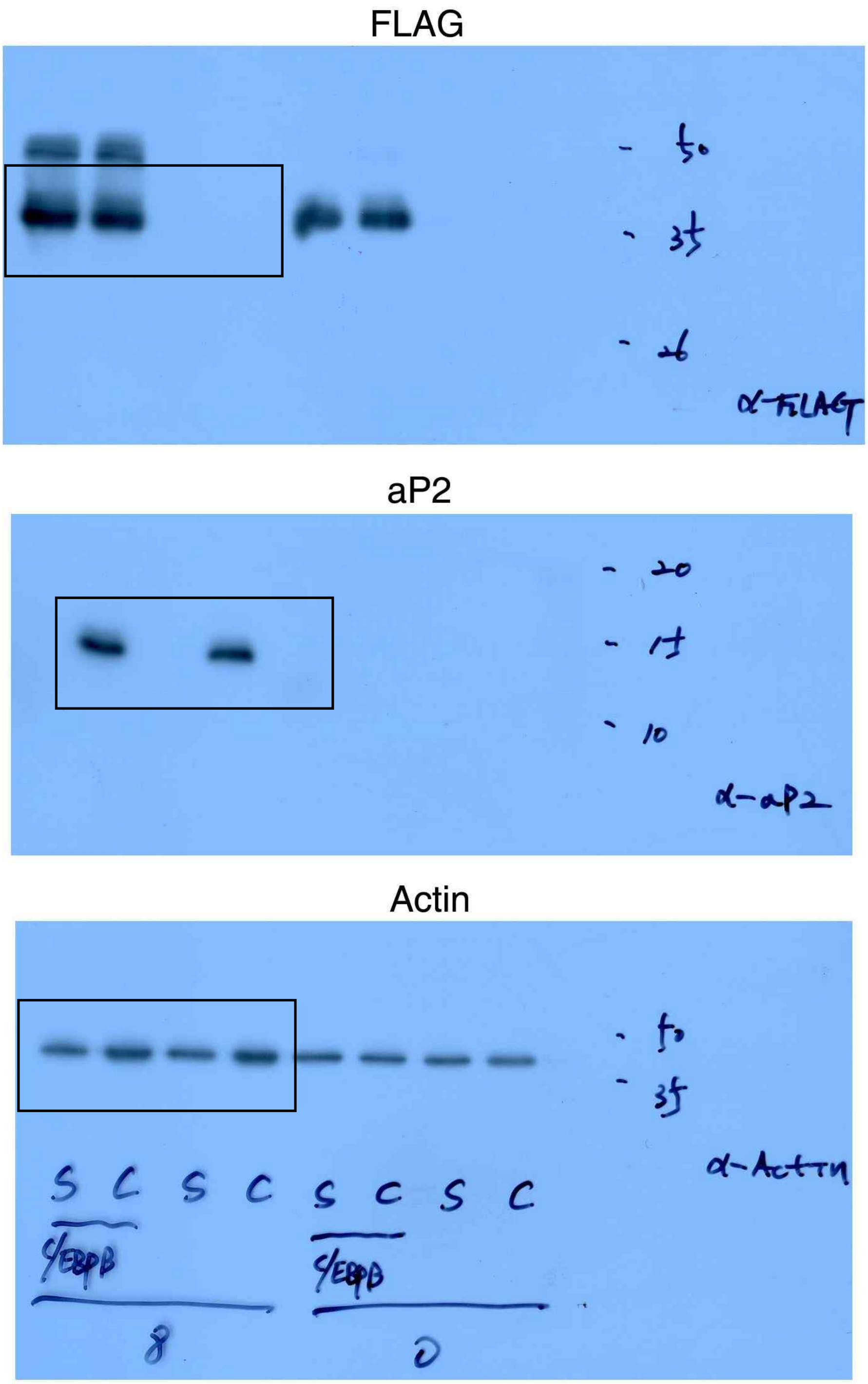

Fig. 2b

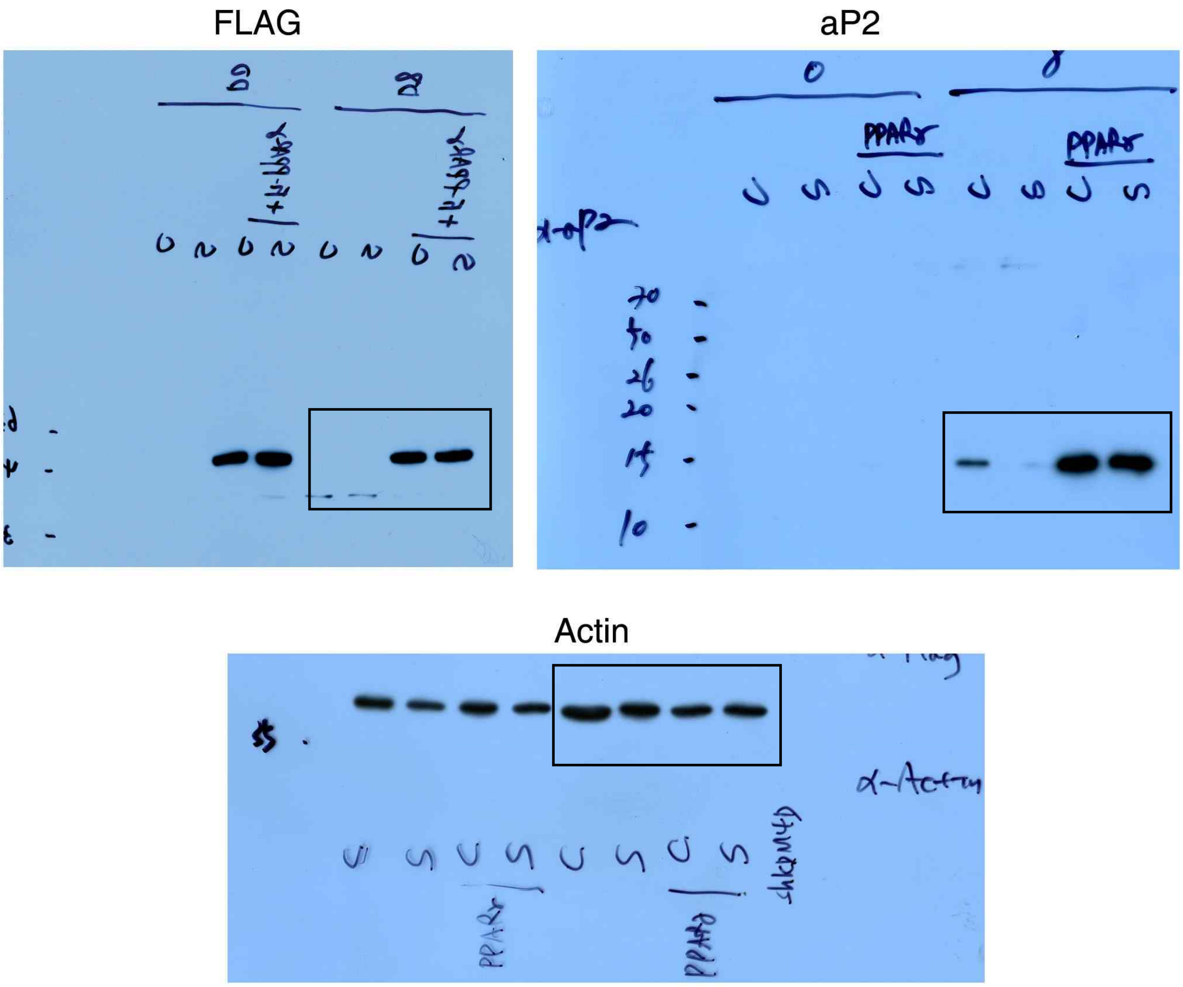

Fig. 2c

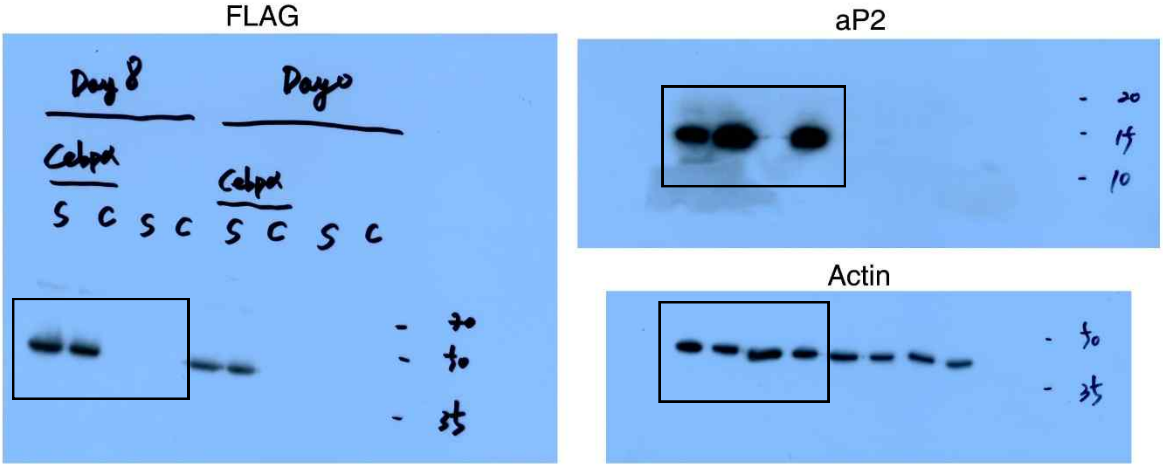

Fig. 3b and 3c

For Fig. 3B

FLAG

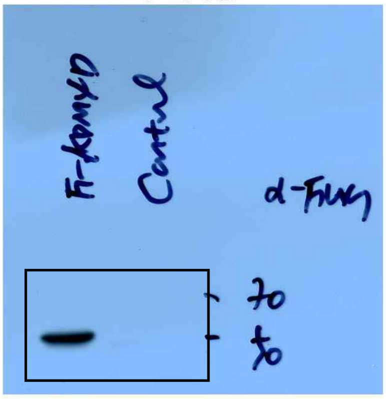

NFIB

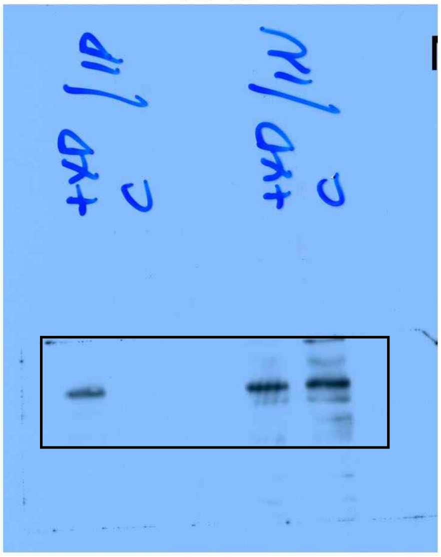

For Fig. 3C

FLAG and KDM4D (Input)

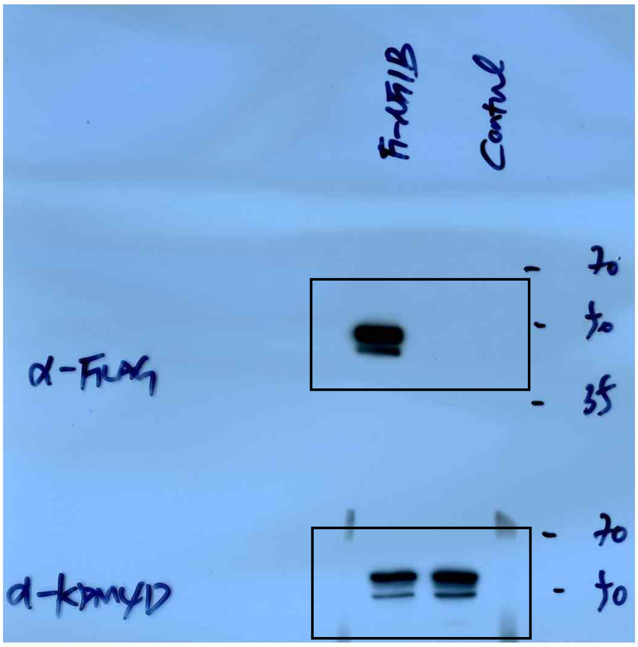

FLAG and KDM4D (IP)

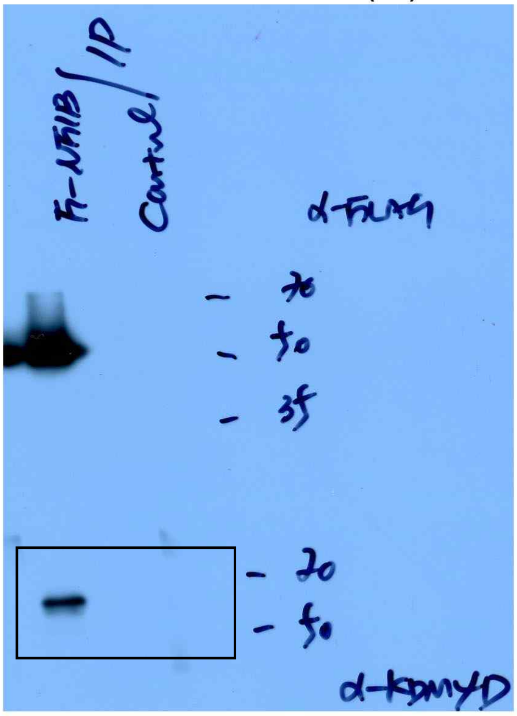

Fig. 3d and 3e

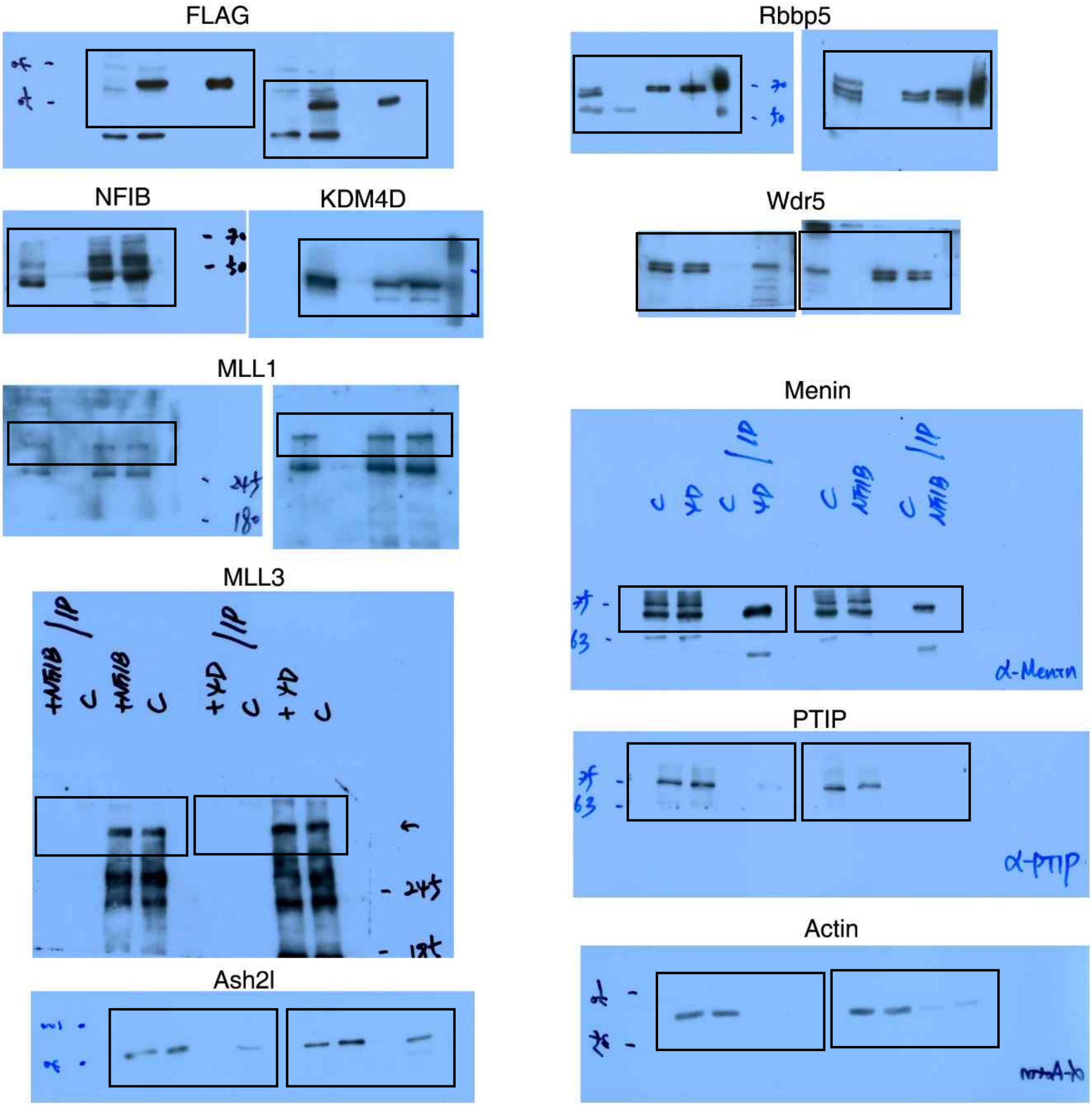

Fig. 3f

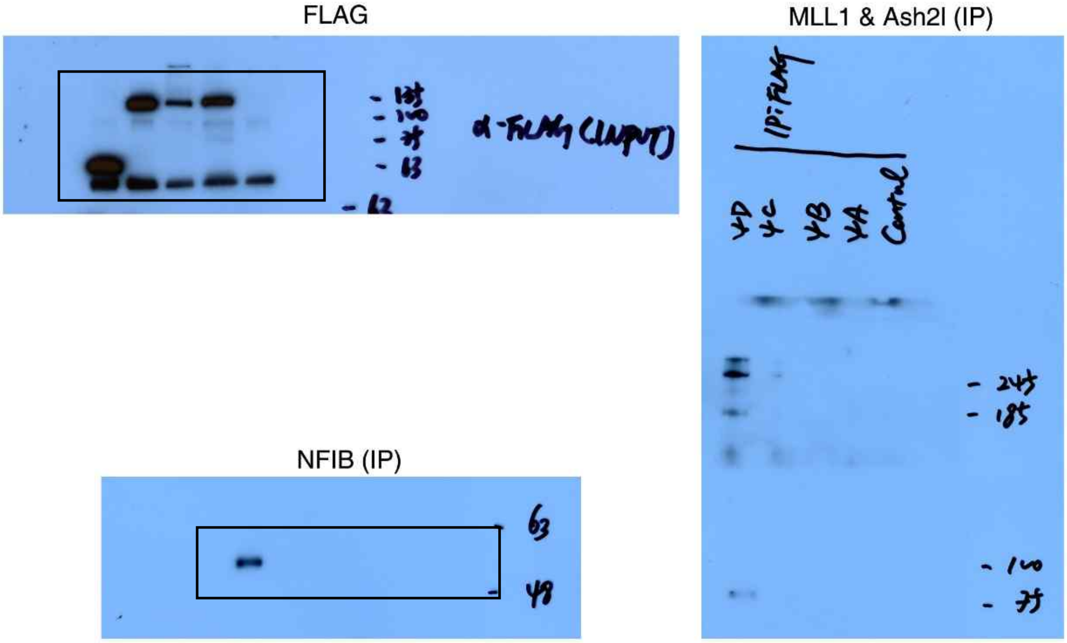

Fig. 3h

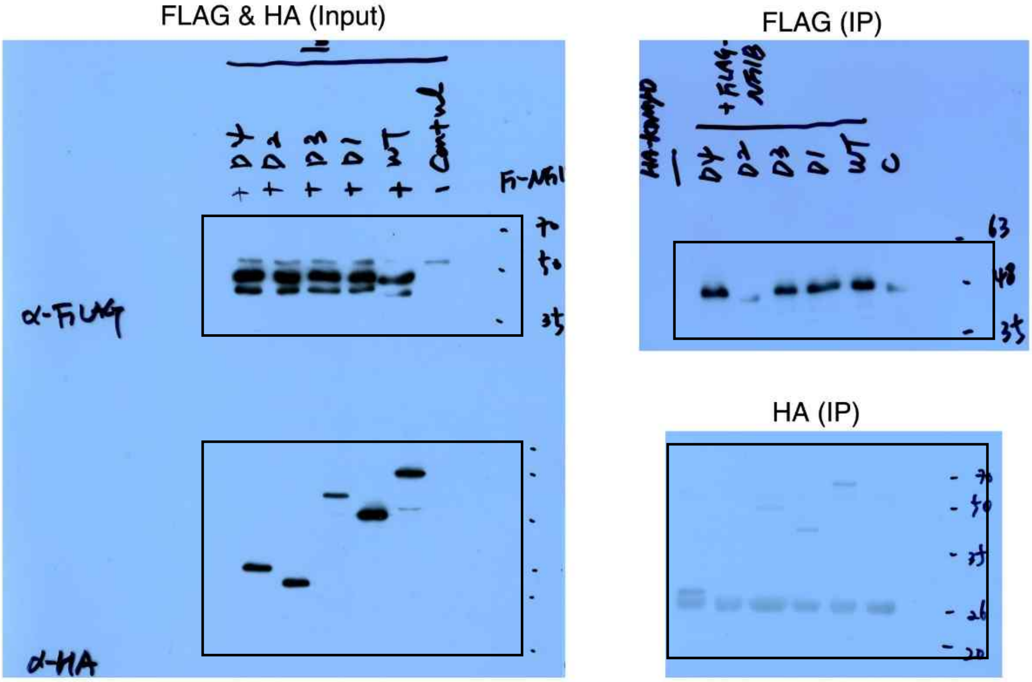

Fig. 3i

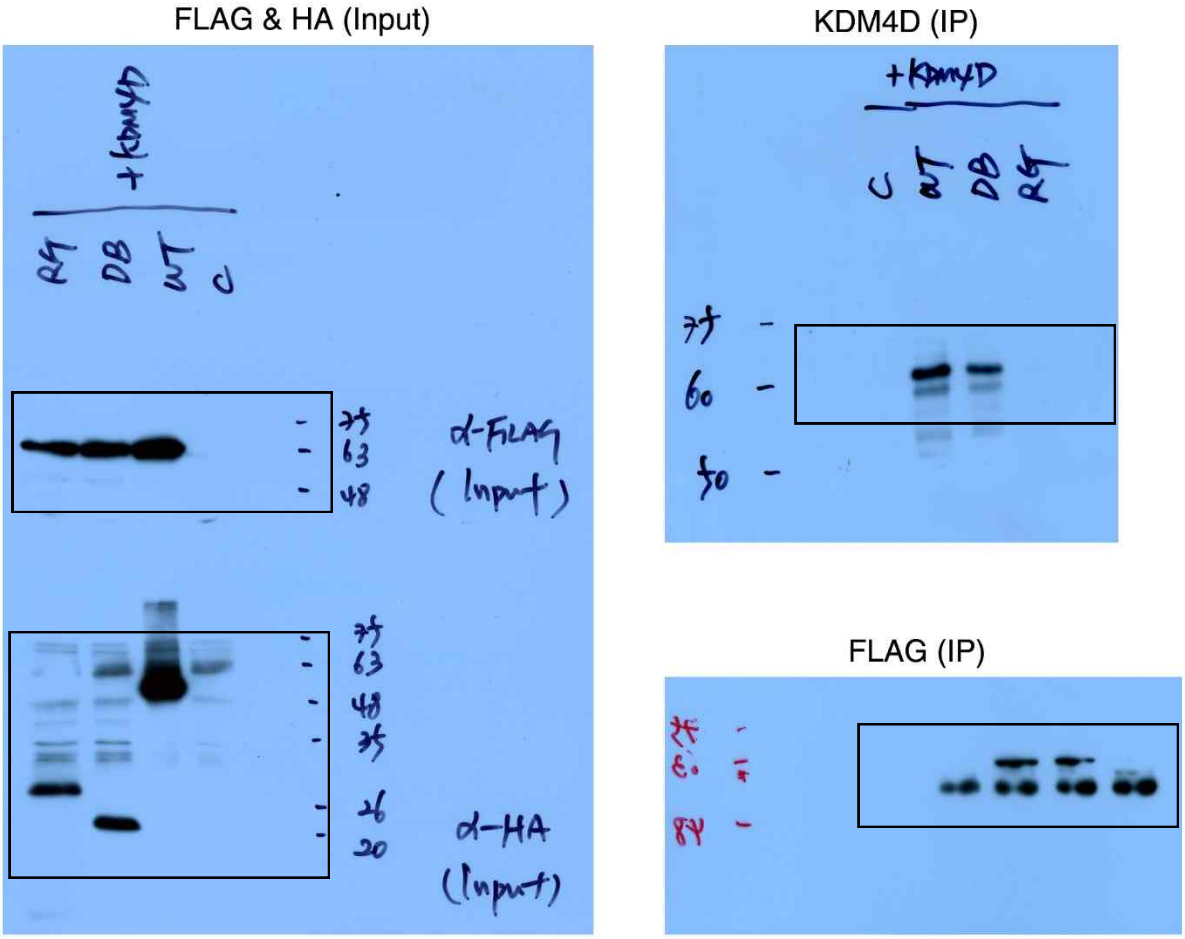

Fig. 5a, 5d, and 5g

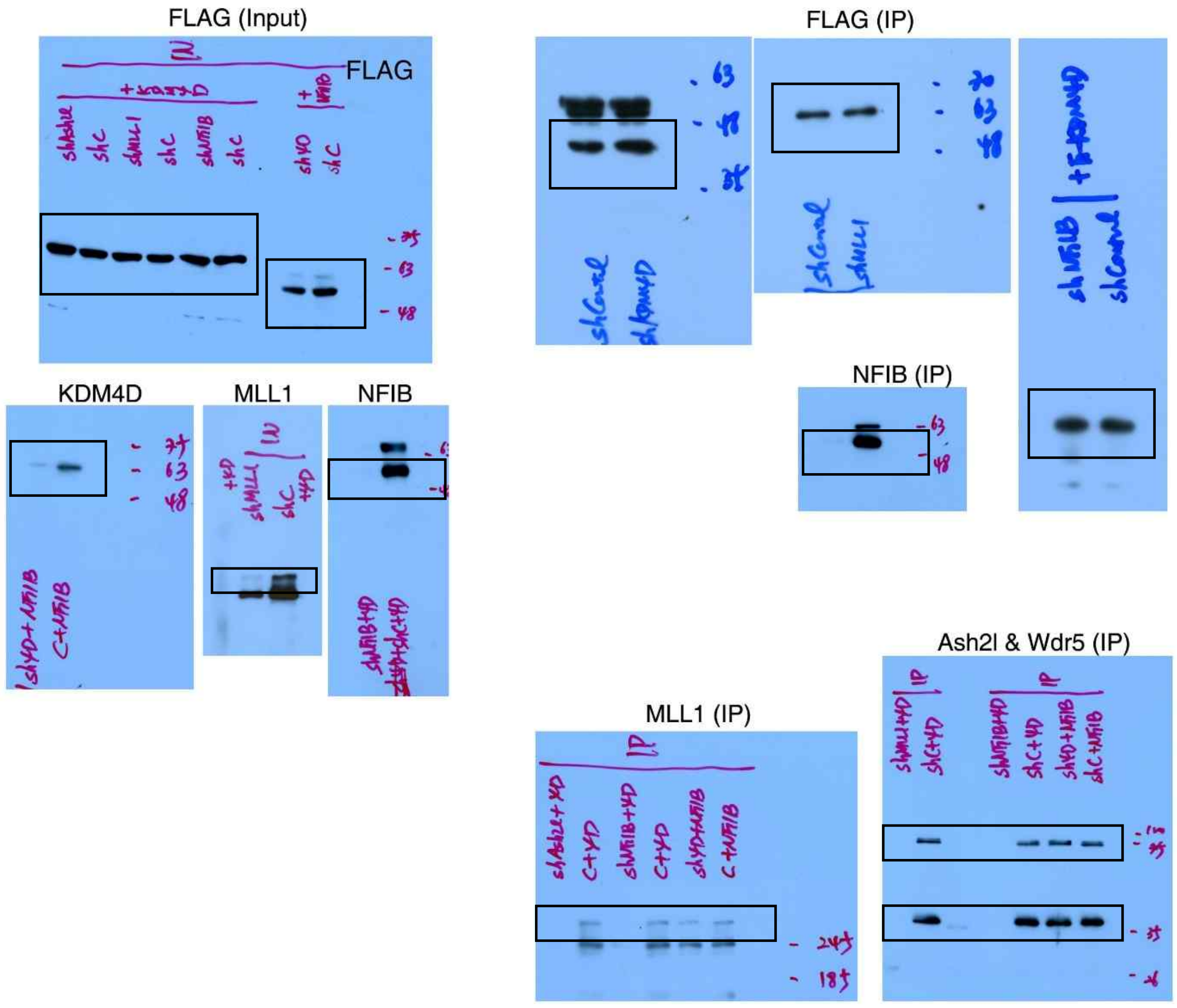

References

[1] Choi, J. H., Song, Y. J. & Lee, H. The histone demethylase KDM4B interacts with MyoD to regulate myogenic differentiation in C2C12 myoblast cells. Biochem. Biophys. Res. Commun. 456, 872–878 (2015).

[2] Hiraike, Y. et al. NFIA co-localizes with PPARγ and transcriptionally controls the brown fat gene program. Nat. Cell Biol. 19, 1081–1092 (2017).

[3] Lin, N. et al. An evolutionarily conserved long noncoding RNA TUNA controls pluripotency and neural lineage commitment. Mol. Cell 53, 1067 (2014).

[4] Gurung, R. & Parnaik, V. K. Cyclin D3 promotes myogenic differentiation and Pax7 transcription. J. Cell. Biochem. 113, 209–219 (2012).

[5] Pei, H., Yao, Y., Yang, Y., Liao, K. & Wu, J.-R. Krüppel-like factor KLF9 regulates PPARγ transactivation at the middle stage of adipogenesis. Cell Death Differ. 18, 315–327 (2011).

[6] Molchadsky, A. et al. P53 is required for brown adipogenic differentiation and has a protective role against diet-induced obesity. Cell Death Differ. 20, 774–783 (2013).

[7] Zhi, H. H., Reardon, C. A. & Mazzone, T. Endogenous ApoE expression modulates adipocyte triglyceride content and turnover. Diabetes 55, 3394–3402 (2006).

[8] Park, Y. K. et al. Hypoxia-inducible factor-2α-dependent hypoxic induction of Wnt10b expression in adipogenic cells. J. Biol. Chem. 288, 26311–26322 (2013).

[9] Yi, S. A. et al. S6K1 phosphorylation of H2B mediates EZH2 trimethylation of H3: a determinant of early adipogenesis. Mol. Cell 62, 443–452 (2016).

[10] Chikka, M. R., McCabe, D. D., Tyra, H. M. & Rutkowski, D. T. C/EBP homologous protein (CHOP) contributes to suppression of metabolic genes during endoplasmic reticulum stress in the liver. J. Biol. Chem. 288, 4405–4415 (2013).

[11] Birsoy, K., Chen, Z. & Friedman, J. Transcriptional regulation of adipogenesis by KLF4. Cell Metab. 7, 339–347 (2008).

[12] Yoo, E. J., Chung, J.-J., Choe, S. S., Kim, K. H. & Kim, J. B. Down-regulation of histone deacetylases stimulates adipocyte differentiation. J. Biol. Chem. 281, 6608–6615 (2006).
